# A randomized controlled trial to evaluate the effect of influenza vaccination and probiotic supplementation on immune response and incidence of influenza-like illness in an elderly population in Indonesia

**DOI:** 10.1371/journal.pone.0250234

**Published:** 2021-12-16

**Authors:** Sukamto Koesnoe, Nuning Masjkuri, Asri Adisasmita, Samsuridjal Djauzi, Cissy Kartasasmita, Julitasari Sundoro, Mardiati Nadjib, Mondastri Korib, Alisa Nurul Muthia, Virly Nanda Muzellina, Ummu Habibah, Saskia Aziza Nursyirwan, Kristoforus Hendra Djaya, Novilia Sjafri Bachtiar, Rini Mulia Sari

**Affiliations:** 1 Division of Allergy and Immunology, Department of Internal Medicine, dr. Cipto Mangunkusumo Hospital, Jakarta, Indonesia; 2 Division of Allergy and Immunology, Department of Internal Medicine, Faculty of Medicine University of Indonesia, Jakarta, Indonesia; 3 Department of Epidemiology, Faculty of Public Health University of Indonesia Jakarta, Jakarta, Indonesia; 4 Department of Child Health, Faculty of Medicine, Padjadjaran University, Hasan Sadikin General Hospital, Bandung, Indonesia; 5 Indonesian Technical Advisory Group on Immunization, Jakarta, Indonesia; 6 Department of Health Policy and Administration, Faculty of Public Health University of Indonesia, Jakarta, Indonesia; 7 Indonesian Society of Internal Medicine, Jakarta, Indonesia; 8 Division of Gastroenterology, Depertment of Internal Medicine, dr. Cipto Mangunkusomo Hospital, Jakarta, Indonesia; 9 Division of Gastroenterology, Depertment of Internal Medicine, Faculty of Medicine University of Indonesia, Jakarta, Indonesia; 10 PT. Bio Farma, Bandung, Indonesia; University of California San Diego, UNITED STATES

## Abstract

**Aim:**

To investigate the effect of influenza vaccination with or without probiotic supplementation on the immune response and incidence of influenza-like illness (ILI) in the elderly.

**Methods:**

A randomized double-blind, placebo-controlled trial with a modified factorial design was conducted in 554 healthy elderly subjects aged 67 ± 5.6 (ranging from 60–90) years old in the Primary Health Care Center (Puskesmas area) of the Pulo Gadung District East Jakarta. Subjects received either a trivalent influenza vaccine or placebo at the start of the study, and a probiotic supplement (*Lactobacillus helveticus* R0052 and *Lactobacillus rhamnosus* R0011) or a placebo for 6 months. Subjects were randomly assigned into four intervention groups: influenza vaccine and probiotics (n = 141), influenza vaccine and placebo (n = 136), placebo and probiotics (n = 140), and both placebo (n = 137). The primary outcome was ILI incidence within 6 months. The secondary outcomes were seroprotection and seroconversion rates at 1, 4, and 6 months after administering the interventions.

**Results:**

This study showed that the trivalent influenza vaccine increased seroprotection (RR 3.6 [95%CI 2.92–4.47]; p<0.010) and seroconversion (RR 29.8 [95%CI 11.1–79.5]; p<0.010) rates 1 month after vaccination in elderly people while the probiotic supplement did not alter influenza antibody titers (p = 1.000 and p = 0.210). The relative ILI incidence risk was similar between vaccinated and non-vaccinated groups, as well as in the probiotic group compared to the non-probiotic group.

**Conclusion:**

The tested trivalent influenza vaccine significantly induced seroprotection and seroconversion in the vaccinated subjects, while probiotics administration did not influence these parameters. Vaccinated individuals displayed a similarly low ILI incidence as those in the Control Group. However, the observed trend towards a reduction of ILI incidence with probiotics supplementation warrants further assessments in a larger, at-risk population.

**Clinical trial registry number:**

NCT03695432.

## Introduction

Influenza is a major cause of mortality and morbidity worldwide [1]. Indeed, influenza viruses can cause only minimal symptoms, but also can lead to severe and lethal complications [2]. In general, influenza virus infections result in *Acute Respiratory Illness* (ARI). However, because ARI symptoms can also be caused by other infectious agents and are not specific to influenza viruses, this set of symptoms is referred to as Influenza-Like Illness (ILI) [3, 4]. In Indonesia, there is currently no available report on the prevalence of this disease. Based on symptoms used to define ARIs, prevalence is estimated at 25% [5]. Various studies have shown that influenza viruses and Respiratory Syncytial Virus (RSV) are often associated with acute respiratory disease requiring hospitalization, especially in the elderly population and patients with previous chronic disease [1, 6]. This is why individuals aged 65 years or older are considered among the most vulnerable groups, representing 90% of the reported cases of influenza-related complications.

Vaccination is considered as a primary preventive method in the management of influenza [7]. The efficacy of a vaccine at preventing disease can be inferred based on its efficacy and effectiveness at inducing seroconversion, conferring seroprotection, and reducing ILI incidence [8]. However, clinical studies on the effectiveness and efficacy of influenza vaccines in elderly populations have generated contradictory results [9].

Immunosenescence, which refers to the process of immune system aging that is reflected by an increased incidence of infections in the elderly, has been proposed as the cause underlying the reduced immunization response to vaccines observed in the elderly population. A new strategy is needed to improve the effectiveness of influenza vaccines in the elderly, either by improving the individuals’ immune response or vaccine formulations [10]. In this study, we explore whether probiotics can improve the immune response triggered by a trivalent influenza vaccine in the elderly, and reduce the incidence of ILI in this population.

## Materials and methods

This study was a randomized, double-blind, placebo-controlled trial with a factorial design comparing the efficacy of two interventions, influenza vaccines and probiotics, at decreasing the risk of ILI in the elderly. The protocol of this study was approved by the Research Ethics Board of the Faculty of Public Health, University of Indonesia. The study protocol has also been registered in the Clinicaltrials.gov Registry, with the clinical trial registry number NCT03695432. There was a non-trial-related technical issue causing the delay in registering the study, that it was performed later after the subject enrollment started.

Eligible participants were randomized into four intervention groups: influenza vaccine + probiotics; influenza vaccine + placebo; placebo + probiotics; and both placebo. This study was conducted in the entire Pulo Gadung District, East Jakarta, between April and December 2015, which was the period encompassing flu season. At the beginning of this research, the dominant strain available was Strain B (lineage not determined), meanwhile Strain A(H1N1)pdm09 predominated later on. And the strains for the vaccines were A/California/7/2009(H1N1)pdm09-like virus, A/Texas/50/2012(H3N2)-like virus, dan B/Massachusetts/2/2012-like virus.

Eligible subjects were healthy adults aged ≥ 60 years who presented themselves to vaccination and health education activities in various Primary Health Care Center (Puskesmas) of the East Jakarta district. In order to be enrolled in the study, potential participants had to have a BMI score between 17.5 and 29.9, and demonstrate a healthy mental state (MMSE score 28–30). Exclusion criteria were: contraindications to influenza vaccination, undergoing an immunomodulatory treatment in the past four weeks, immunosuppressant therapy, taking corticosteroids such as prednisone ≥ 20 mg/d for more than two weeks or for less than three months before the study. Potential participants were also excluded if they had previous influenza vaccination less than one year before the study, or were consuming probiotics (either manufactured or natural products) for more than seven days.

Screening was performed based on convenience sampling. Written informed consent was obtained from each subject prior to the trial-related screening. At baseline, blood samples were collected to measure basal influenza antibody levels. Then, eligible subjects were assigned into any of the four intervention groups, according to the randomization code provided by a contract research organization as the third party. The randomization code was generated by utilizing the Microsoft Excel^®^ software. Subjects received the trivalent inactivated influenza vaccine (Flubio^®^, Biofarma, Bandung) or placebo (NaCl 0.9% solution) at the study initiation visit (month 0), with a supply of either Lacidofil^®^ (*Lactobacillus acidophilus* R0052 and *Lactobacillus rhamnosus* R0011) or placebo. Therefore, all participants received similar interventions, that were the vaccine injection (or its placebo) and the oral probiotic capsules (or its placebo), in order to keep the investigator team, including the care providers and those assessed the outcomes, all subjects, as well as the laboratory personnel, blinded to the intervention allocation.

Follow-up visits were scheduled at 1, 4, and 6 months post-vaccination. The primary outcome was ILI incidence within 6 months. The secondary outcomes were seroprotection and seroconversion rates at 1, 4, and 6 months after administering the interventions. Compliance with probiotic supplementation was assessed by the study personnel of the Integrated Health Service Center (Posyandu), based on participant’s self-reported data.

To determine the efficiency of the influenza vaccine, the number of participants showing seroconversion or seroprotection was assessed. Participants with a baseline antibody titers <1:10 were considered seronegative at baseline, and a post-vaccination titer ≥ 1:40 was used to define seroprotection. A 4-times increase in the antibody titers after vaccination was required to infer seroconversion in those with a final titer having reached or exceeded 1:40.

Sample size was estimated based on projected ILI rates for the study target period. Our hypothesis was that the ILI incidence would be lower in the vaccinated group than in the corresponding placebo group. Similarly, we anticipated ILI incidence to be lower in the probiotics group than in the corresponding placebo group. Previously reported ILI rates in non-vaccinated elderly patients was 26.4% [4], and in vaccinated and probiotics-treated elderly individuals were 15.84% and 11.88%, respectively [4]. The ILI prevention effectiveness of the influenza vaccine was approximately 40% [11], and of probiotics was 55% [12]. Assuming a similar chance (π) to get ILI for all subjects in all groups at baseline (pre-vaccination), and using a significance level (α) of 0.05, and a statistical power (1–β) of 80%, the minimum required sample size was 266 subjects for each group, estimated using the Freedman’s Equation below:

$$n=\frac{1}{\left(\pi_{c}+\;\pi_{t}\right)}\;{\left(\frac{\theta +1}{\theta -1}\right)}^{2}\;{\left(Z_{1-\alpha }+\;Z_{1-\beta }\right)}^{2}$$

where θ refer to the expected hazard ratio, and π is the chance to get ILI at baseline (pre-vaccination).

Therefore, for this two-by-two factorial design study, a total of 592 subjects were required, anticipating a drop-out rate of 10%. Each factorial group would require 148 subjects.

The rates of seroprotection and seroconversion as well as the ILI incidence between-group were statistically analyzed using chi-square test. While within-group analysis on seroprotection rate of each time-point after vaccination compared to the baseline (Pre-vaccination) was performed using cochran-Q test. All statistical analysis was performed at a significance level (α) of 0.05, 2-tailed. The analysis was performed using a statistical software Microsoft SPSS version 24.

## Results

**Fig 1:**
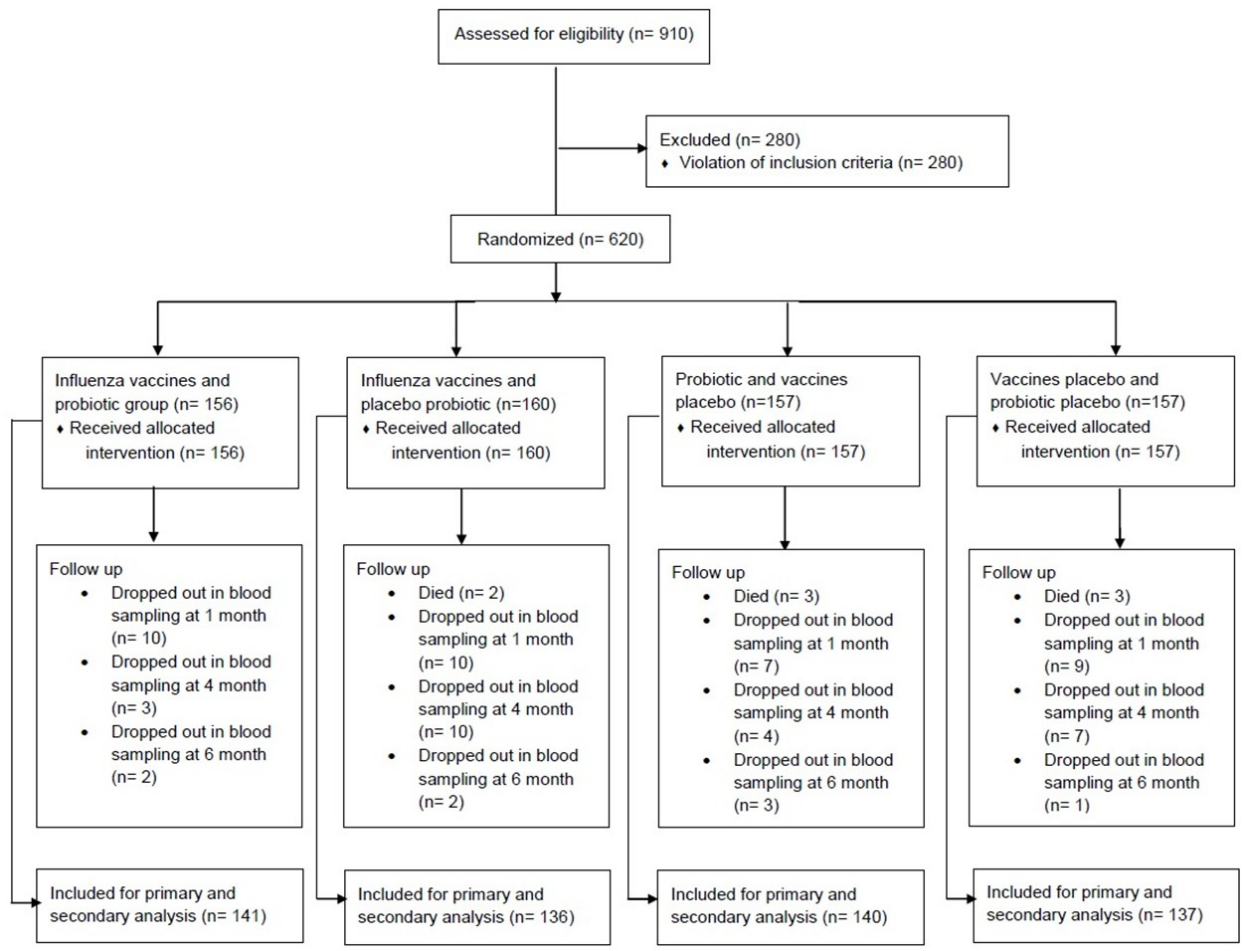
Subject disposition.

A total of 910 participants were screened between April and June 2015; of them, 280 were excluded for not meeting the inclusion criteria and the remaining 620 subjects were included and randomized. Of the remaining subjects, 554 completed the study and were included in subsequent analyses. Two primary interventions were studied: influenza vaccines and probiotic supplementation (Fig 1). Patient characteristics at baseline were similar between both interventions (Table 1) and between study groups (Table 2). Statistical analyses revealed no significant interaction between groups receiving vaccines and those receiving probiotics (OR 0.924, [95% CI 0.606–1.407]; p = 0.712).

**Table 1:** Baseline characteristics of all participants included in the analyses, clustered by intervention.

Characteristics	Influenza Vaccine Intervention^a^			Probiotic Intervention^b^		
	Vaccine (n = 277)	Non-Vaccine (n = 277)	p value	Probiotic (n = 281)	Non-Probiotic (n = 273)	p value
**Age group**						
60–65 (n = 242[43.7])	130 (49.6)	112 (40.4)		123 (43.8)	119 (43.6)	
66–70 (n = 144[26])	67 (24.2)	77 (27.8)		69 (24.6)	75 (27.5)	
71–75 (n = 112[20.2])	53 (19.1)	59 (21.3)	0.573	60 (21.4)	52 (19.0)	0.649
76–80 (n = 39[7.0])	20 (7.2)	19 (6.9)		18 (6.4)	21 (7.7)	
> 80 (n = 17[3.1])	7 (2.5)	10 (3.6)		11 (3.9)	6 (2.2)	
**Gender**						
Male (n = 198[35.7])	97 (35)	101 (36.5)	0.732	99 (35.2)	99 (36.3)	0.800
Female (n = 356[64.3])	180 (65)	176 (63.5)		182 (64.8)	174 (63.7)	
**Marital Status**						
Unmarried (n = 3[0.5])	3 (1.0)	0 (0)		1 (0.4)	2 (0.7)	
Divorced (n = 246[44.4])	150 (54.2)	155 (56)	0.212	157 (55.9)	148 (54.2)	0.785
Married (n = 305[55.1])	124 (44.8)	122 (44)		123 (43.7)	123 (45.1)	
**Education**						
High (n = 227[41])	110 (39.7)	117 (42.2)	0.545	103 (36.7)	124 (45.4)	0.036
Low (n = 327[59])	167 (60.3)	160 (57.8)		178 (63.3)	149 (54.6)	
**Number of Residents at Home**						
Less than 4 (n = 271[48.9])	139 (50.2)	132 (47.7)	0.552	131 (46.6)	140 (51.3)	0.272
More than 4 (n = 283[51.1])	138 (49.8)	145 (52.3)		150 (53.4)	133 (48.7)	
**Nutritional Status**						
Normal Body weight (n = 172[31.0])	83 (30)	89 (32.1)	0.607	84 (29.9)	88 (32.2)	0.740
Overweight (n = 275[49.6])	136 (49.1)	139 (50.2)		144 (51.2)	131 (48)	
Obese (n = 107[19.3])	58 (20.9)	49 (17.7)		53 (18.9)	54 (19.8)	
**Hypertension**						
No (n = 247[44.6])	170 (61.4)	137 (49.5)	0.005	153 (54.4)	154 (56.4)	0.642
Yes (n = 307[55.4])	107 (38.6)	140(50.5)		128 (45.6)	119 (43.6)	
**Diabetes mellitus**						
No (n = 442[79.8])	220 (79.4)	222 (80.1)	0.832	220 (78.3)	222 (81.3)	
Yes (n = 112[20.2])	57 (20.6)	55 (19.9)		61 (21.7)	51 (18.7)	0.375
**Cardiovascular**						
No (n = 50[9.0])	253 (91.3)	251 (90.6)	0.767	263 (93.6)	241 (88.3)	
Yes (n = 504[91])	24 (8.7)	26 (9.4)		18(6.4)	32 (11.7)	0.029
**Cerebrovascular**						
No (n = 540[97.5])	7 (2.5)	270 (97.5)	1.00	275 (97.9)	265 (97.1)	
Yes (n = 14[2.5])	7(2.5)	270 (97.5)		6 (2.1)	8 (2.9)	0.551
**Chronic Pulmonary Disease**						
No (n = 524[94.6])	263 (94.9)	261 (94.2)	0.707	267 (95.0)	257 (94.1)	
Yes (n = 30[5.4])	14 (5.1)	16 (5.8)		14 (5.0)	16 (5.9)	0.648
**Exercise**						
≥3x/week min. 30 min (n = 113[20.4]	55 (19.9)	58 (20.9)	0.752	58 (20.6)	55 (20.1)	0.885
< 3x/week min. 30 min (n = 441[79.6])	222 (80.1)	219 (79.1)		223 (79.4)	218 (79.9)	
**Smoking**						
Non-smoker (n = 391[70.6])	197 (71.1)	194 (70)	0,780	203 (72.2)	188 (68.9)	0.383
Smoker (n = 163[29.4])	80 (28.9)	83 (30)		78 (27.8)	85 (31.1)	
**Vaccination History**						
Yes (n = 19[3.4])	12 (4.3)	7 (2.5)	0.243	8 (2.8)	11 (4.0)	0.445
No (n = 535 [96.6])	265 (95.7)	270 (97.5)		273 (97.2)	262(96.0)	
**Dependency Level**						
Independence (n = 513[92.6])	253 (91.3)	260 (93.9)	0.256	261 (92.9)	252 (92.3)	0.796
Moderate dependency (n = 41[7.4])	24 (8.7)	17 (6.1)		20 (7.1)	21 (7.7)	
**Frailty Index**						
Non-*frail* (n = 202[36.5])	110 (39.7)	92 (33.2)	0.133	100 (35.6)	102 (37.4)	0.730
*Frail* (n = 352[63.5])	167 (60.3)	185 (66.8)		181 (64.4)	171 (62.6)	
**Depression Level**						
Non-depressed (n = 458[82.7])	227 (81.9)	231 (83.4)	0.736	232 (82.6)	226 (82.8)	1.00
Depressed (n = 96[17.3])	50 (18.1)	46 (16.6)		49 (17.4)	47 (17.2)	
**Basal Seroprotection level**						
Positive (n = 159[28.7])	84 (30.3)	75 (27.1)	0.398	80 (28.5)	79 (28.9)	0.903
Negative (n = 395[71.3])	193 (69.7)	202 (72.9)		201 (71.5)	194 (71.1)	
	67 (24.2)	77 (27.8)		69 (24.6)	75 (27.5)	

All data is presented as n (%).

^**a**^ All participants included in the analyses (n = 554), clustered according to the vaccine intervention (n = 277) and corresponding placebo (n = 277), regardless of the supplemented product (probiotics or placebo).

^**b**^ All participants included in the analyses (n = 554), clustered according to the probiotics intervention (n = 281) and corresponding placebo (n = 273), regardless of the vaccination received (vaccine or placebo).

**Table 2:** Participant characteristics in the four intervention groups.

Variable	Category n (%)	Intervention								
		Influenza Vaccine and Probiotics (n = 141)		Influenza Vaccine and Placebo (n = 136)		Placebo and Probiotics (n = 140)		Both Placebo (n = 137)		P Value
		N	%	N	%	N	%	N	%	
	Median	66 (60–85)		66 (60–86)		67 (60–90)		67 (60–85)		
**Age**	60–65 (n = 242[43.7])	66	46.8	64	47.1	57	40.7	55	46.8	0.838
	66–70 (n = 144[26])	31	22.0	36	26.5	38	27.1	39	22.0	
	71–75 (n = 112[20.2])	29	2.6	24	17.6	31	22.1	28	2.6	
	76–80 (n = 39[7.0])	11	7.8	9	6.6	7	5.0	12	7.8	
	> 80 (n = 17[3.1])	4	2.8	3	2.2	7	5.0	3	2.8	
**Gender**	Men (n = 198[35.7])	45	31.9	52	38.2	54	38.6	47	31.9	0.598
	Women (n = 356[64.3])	96	68.1	84	61.8	86	61.4	90	68.1	
**Status**	Not married (n = 3[0.5])	1	0.7	2	1.5	0	0	0	0.7	0.675
	Divorced ([246[44.4])	63	44.7	61	44.9	60	42.9	62	44.7	
	Married ([305[55.1])	77	54.6	73	53.7	80	57.1	75	54.6	
**Education**	High (n = 227[41])	46	32.6	64	47.1	57	40.7	60	32.6	0.086
	Low (n = 305[55.1])	95	67.4	72	52.9	83	59.3	77	67.4	
**Number of Resident**	Less than 4 n = 271[48.9])	76	53.9	62	45.6	74	52.9	71	53.9	0.513
	More than 4 (n = 283[51.1])	65	46.1	74	54.4	66	47.1	66	46.1	
**Nutritional Status**	Normal (n = 172[31.0])	45	31.9	38	27.9	39	27.9	50	31.9	0.603
	Overweight (n = 275[49.6])	67	47.5	69	50.7	77	55.0	62	47.5	
	Obese (n = 107[19.3]	29	20.6	29	21.3	24	17.1	25	20.6	
**Hypertension**	No (n = 247[44.6])	82	58.2	88	64.6	71	50.7	66	48.2	0.025
	Yes (n = 307[55.4])	59	41.8	48	35.3	69	49.3	71	51.8	
**Diabetes Mellitus**	No (n = 442[79.8])	109	77.3	111	81.6	111	79.3	111	81	0.808
	Yes (n = 112[20.2])	32	22.7	25	18.4	29	20.7	26	19	
**Cardiovascular**	No (n = 50[9.0])	133	94.3	120	88.2	130	92.9	121	88.3	0.175
	Yes (n = 504 [91]	8	5.7	16	11.8	10	7.1	16	11.7	
**Cerebrovascular**	No (n = 540[97.5])	139	98.6	131	96.3	136	97.1	134	97.8	0.669
	Yes (n = 14[2.5])	2	1.4	5	3.8	4	2.9	3	2.2	
**Lungs**	No (n = 524[94.6])	134	95	129	94.9	133	95	128	93.4	0.924
	Yes (n = 30[5.4])	7	5.0	7	5.1	7	5.0	9	6.6	
**Physical Exercise**	≥ 3x/week min. 30 minutes (n = 113[20.4])	27	19.1	28	20.6	31	22.1	27	19.7	0.932
	< 30x/week min. 30 minutes (n = 441[79.6])	114	80.9	108	79.4	109	77.9	110	80.3	
**Smoking History**	Not smoking (n = 391[70.6])	106	75.2	91	66.9	97	69.3	97	70.8	0.488
	Smoking (n = 163[29.4])	35	24.8	45	33.1	43	30.7	40	29.2	
**Vaccine History**	Vaccines (n = 19[3.4])	6	4.3	6	4.4	2	1.4	5	3.6	0.494
	Non-vaccines (n = 535[96.6])	135	95.7	130	95.6	138	98.6	132	96.4	
**Dependency**	Independent (n = 513[92.6])	130	92.2	123	90.4	131	93.6	129	94.2	0.651
	Dependent (n = 41[7.4])	11	7.8	13	9.6	9	6.4	8	5.8	
**Frailty Index**	Non-frail (n = 202[36.5])	90	63.8	77	56.6	91	65	94	68.6	0.215
	Frail (n = 352[63.5])	51	36.2	59	43.4	49	35	43	31.4	
**Depression Level**	Not depressed (n = 458[82.7])	116	82.3	111	81.6	116	82.9	115	83.9	0.964
	Depressed (n = 96[17.3])	25	17.7	25	18.4	24	17.1	22	16.1	
**Seroprotection**	Seroprotection (n = 159[28.7])	45	31.9	39	28.7	35	25	40	29.2	0.645
	No seroprotection (n = 395[71.3])	96	68.1	97	71.3	105	75	97	70.8	

The distribution of participants demonstrating seroconversion and seroprotection are presented in Table 4. There was a significant increase in post-vaccination seroprotection in groups receiving vaccines with probiotics and without probiotics at 1, 4, and 6 months, but it was not significant for groups who did not get vaccination (Table 3). Thus, in the proportion of seroconversion, there was no significant difference between the subjects who received vaccinations and probiotics compared to subjects have only received the vaccine at 1, 4, and 6 months (Tables 4 and 5).

**Table 3:** The proportion of subjects with seroprotective titers at baseline (Pre-vaccination) and at 1, 4, and 6 months after vaccination.

	N		Pre Vaccination (%)	1 month (%)	p value	4 months (%)	p value	6 months (%)	P value
**Influenza vaccine + probiotics**	141	Yes	31.9	92.9	0.035	81.6	0.025	73	0.022
		No	68.1	7.1		18.4		27	
**Influenza vaccine + placebo probiotics**	136	Yes	28.7	92.6	0.040	83.8	0.050	74.3	0.020
		No	71.3	7.4		16.2		25.7	
**Placebo vaccine + probiotics**	140	Yes	25	25.7	1.000	23.4	0.650	22.1	0.610
		No	75	74.3		73.6		77.9	
**Both placebo**	137	Yes	29.2	26.3	0.490	23.4	0.580	19	0.550
		No	70.8	73.7		76.6		81	

Note: p value in this table is for within group comparison of each timepoint with its baseline.

**Table 4:** The proportion of subjects who experience seroconversion after 1, 4, and 6 months following vaccination.

		Seroconversion					
		1 month		4 months		6 months	
		N	%	N	%	N	%
**Influenza vaccine + probiotics (n = 141)**	Yes	65	46.1	35	24.8	19	13.5
	No	76	53.9	106	75.2	122	86.5
**Influenza vaccine + probiotics placebo (n = 136)**	Yes	54	39.7	28	20.6	21	15.4
	No	82	60.3	108	79.4	115	84.6
**Vaccine placebo + probiotics (n = 140)**	Yes	4	2.9	2	1.4	2	1.4
	No	136	97.1	138	98.6	138	98.6
**Both placebo (n = 137)**	Yes	0	0	2	1.5	1	0.7
	No	137	100	135	98.5	136	99.3

**Table 5:** Seroconversion status in the vaccine supplementation + probiotic group compared to the vaccine + placebo group.

		1 month (%)		p value		4 months (%)		p Value		6 months (%)		p Value
	N	Yes	No		N	Yes	No		N	Yes	No	
**Influenza vaccine + probiotics**	65	46.1	53.9		35	24.8	75.2		19	13.5	86.5	
**Influenza vaccine + placebo**	54	39.7	60.3	0.29	28	20.6	79.4	0.21	21	15.4	84.6	0.140

Without considering probiotic supplementation, the relative risk (RR) associated with ILI incidence was similar between participants who received the influenza vaccine and those who received the vaccine placebo (RR = 1.0). When considering probiotic supplementation, the relative ILI incidence risk slightly lower (RR = 0.8) (Table 6). There was no significant difference in the relative ILI incidence risk according to intervention (Table 7).

**Table 6:** Relative ILI incidence risk in vaccine vs non-vaccine intervention groups, and in probiotic vs non-probiotic intervention groups.

	ILI incidence				Total		RR 95% CI	P Value
	ILI		Non-ILI					
	N	%	N	%	N	%		
**Vaccines**	9	3	268	97	277	100	1.0 (0.40–2.48)	1.000
**Non-vaccines**	9	3	268	97	277	100		
**Probiotic**	8	3	273	97	281	100	0.8 (0.31–1.94)	0.800 (0.31–1.94)
**Non-probiotic**	10	4	263	96	273	100		

**Table 7:** Difference in ILI incidence according to intervention.

Intervention	ILI Incidence		Total		RR (95% CI)	P Value
	ILI n(%)	Non-ILI n (%)	N	%		
Influenza vaccine and probiotics	4 (2.8)	137 (97.2)	141	100	0.8 (0.21–2.81)	0.956
Influenza vaccine and placebo	5 (3.7)	131 (96.3)	136	100		
Influenza vaccine and probiotics	4 (2.8)	137 (97.2)	141	100	0.8 (0.21–2.83)	0.702
Both placebo	5 (3.6)	132 (96.4)	137	100		
Placebo and probiotics	4 (2.9)	136 (97.1)	140	100	0.8 (0.21–2.86)	0.710
Both placebo	5 (3.6)	132 (96.4)	137	100		
Influenza vaccine and probiotics	4 (2.8)	137 (97.2)	141	100	1.0 (0.25–3.89)	0.920
Placebo and probiotics	4 (2.9)	136 (97.1)	140	100		
Influenza vaccine and placebo	5 (3.7)	131 (96.3)	136	100	1.0 (0.30–3.40)	0.991
Both placebo	5 (3.6)	132 (96.4)	137	100		
Both placebo	5 (3.6)	132 (96.4)	137	100	1.3 (0.35–4.70)	0.965
Placebo and probiotics	4 (2.9)	136 (97.1)	140	100		

**Fig 2:**
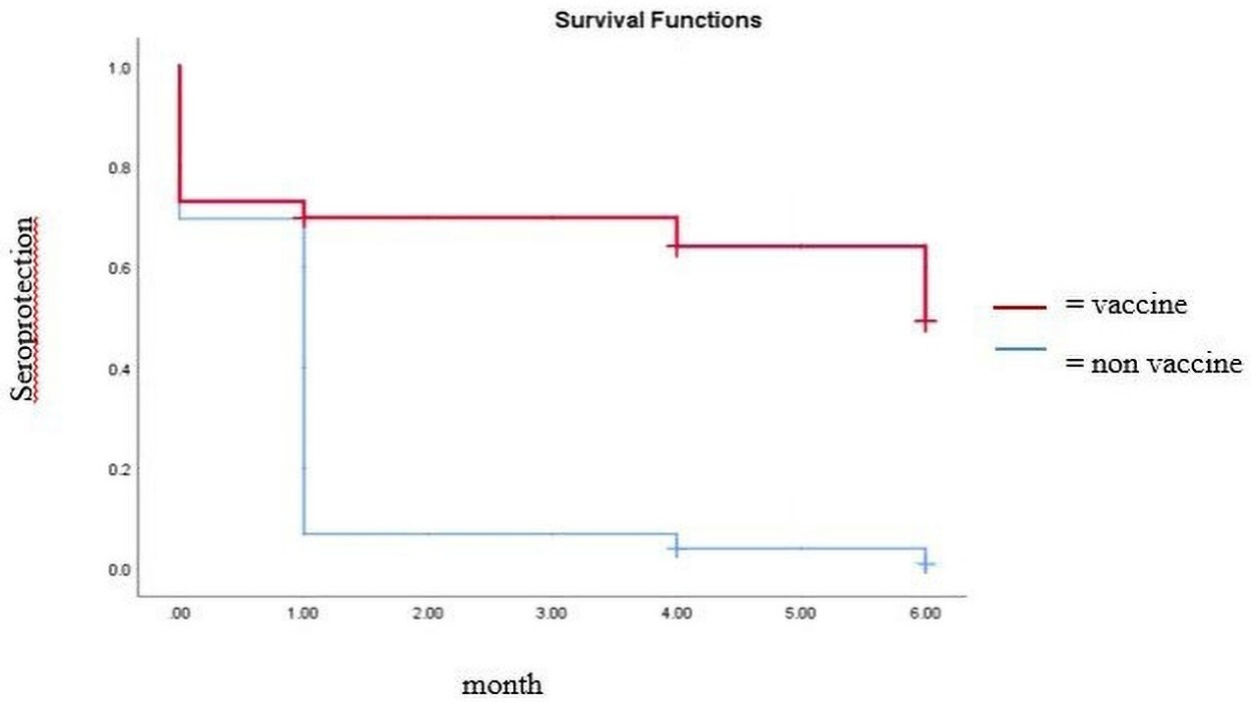
Kaplan-Meier seroprotection diagram.

Statistical analyses showed that the influenza vaccine did not reduce ILI relative risk (RR = 1.0) compared to non-vaccinated groups despite the positive effect of vaccination on the seroprotection status at 1, 4, and 6 months post-vaccination. Kaplan-Meier analysis for the seroprotection (Fig 2) showed a significant difference in the maintenance of the seroprotection for 6 months between those who received the influenza vaccine and those who did not. The geometric mean titers (GMT) of the antibody anti-influenza over months are presented in Table 9. The antibody titers peaked out one month post-vaccination, and then gradually declined toward 6 months post-vaccination. The titer at Month 6 post-vaccination was still higher than that of pre-vaccination.

Of note, there was a non-significant reduction of the relative ILI risk (RR = 0.8) in participants receiving probiotics compared to those not receiving probiotics. However, probiotics administration did not influence the seroprotection and seroconversion status (Table 8).

**Table 8:** ILI incidence, seroprotection, and seroconversion in influenza vaccine intervention (vaccine vs non-vaccine) and in probiotic intervention (probiotic vs non-probiotic).

	Influenza Vaccine				Probiotics			
	Vaccine (n = 277) n (%)	Non-Vaccine (n = 277) n (%)	RR (95% CI)	P Value	Probiotics (n = 281) n (%)	No Probiotics (n = 273) n (%)	RR (95% CI)	P Value
ILI	9 (3.2)	9 (3.2)	1.0 (0.40–2.48)	1.000	8 (2.8)	10 (3.7)	0.8 (0.31–1.940)	0.588
No ILI	268 (96.8)	268 (96.8)			273 (97.2)	263 (96.3)		
Seroprotection 0 month								
Seroprotection	84 (30.3)	75 (27.1)	1.1 (0.86–1.46)	0.398	80 (28.5)	79 (28.9)	1.0 (0.76–1.28)	0.903
No Seroprotection	193 (69.7)	202 (72.9)			201 (71.5)	194 (71.1)		
Seroprotection 1 month								
Seroprotection	257 (92.8)	72 (26)	3.6 (2.92–4.47)	< 0.010	168 (59.4)	163 (59.3)	1.0 (0.87–1.15)	1.000
No Seroprotection	20 (7.2)	205 (74)			115 (40.6)	112 (40.7)		
Seroprotection 4 months								
Seroprotection	229 (82.7)	68 (24.5)	3.3 (2.72–4.17)	< 0.010	151 (53.7)	146 (53.5)	1.0 (0.86–1.17)	1.000
No Seroprotection	48 (17.3)	209 (75.5)			130 (46.3)	127 (46.5)		
Seroprotection 6 months								
Seroprotection	204 (73.6)	57 (20.6)	3.6 (2.81–4.56)	< 0.010	134 (47.7)	127 (46.5)	1.0 (0.86–1.22)	0.849
No Seroprotection	73 (26.4)	220 (79.4)			147 (52.3)	146 (53.5)		
Seroconversion 1 month								
Seroconversion	119 (43)	4 (1.4)	29.8 (11.1–79.5)	< 0.010	69 (24.6)	54 (19.8)	1.2 (0.91–1.7)	0.211
No Seroconversion	158 (57)	273 (98.6)			212 (75.4)	219 (80.2)		
Seroconversion 4 months								
Seroconversion	63 (22.7)	4 (1.4)	15.8 (5.81–42.7)	< 0.010	37 (13.2)	30 (11.0)	1.2 (0.76–1.88)	0.512
No Seroconversion	214 (77.3)	273 (98.6)			244 (86.6)	243 (89.0)		
Seroconversion 6 months								
Seroconversion	40 (14.4)	3 (1.1)	13.3 (4.17–42.6)	< 0.010	21 (7.5)	22 (8.1)	0.9 (0.52–1.65)	0.920
No Seroconversion	237 (85.6)	274 (98.9)			260 (92.5)	251 (91.9)		

## Discussion

In most adults, a baseline level of pre-vaccination antibodies is detectable from ongoing influenza infections or previous vaccinations. Therefore, the humoral immune responses to certain viral strains in younger people are often different than in the elderly. As such, the post-vaccination antibody responses may strongly be affected by the priming process, without strictly representing the ability of the immune system.

The study in Hong Kong by Hui et al [13] also showed a relationship between seroprotection and influenza vaccination. The Hui et al study was based on a population with similar demographic and clinical characteristics as ours. Four weeks after vaccination, the seroprotection rate was 85.9% for H1N1 (OR = 8.1 [95% CI 0.6–47.8] p = 0.115) and 100% for Influenza B (p = 0.500). In our study, the seroprotection status after 1 month post-vaccination was significantly increased (chi-square = 83.101; p<0.010) as shown in Tables 3 and 4.

The outcomes of data analysis of this study in Table 5 show the seroconversion status. There was no significant increase among the intervention groups. Our statistical analyses showed that seroconversion rates were higher in the influenza vaccine + probiotics as well as in the influenza vaccine + placebo groups at the 1 month time point compared to the groups who did not receive the vaccine.

Furthermore, our data suggest that influenza vaccination with this trivalent vaccine was able to adequately stimulate the production of vaccine-specific antibodies at levels sufficient to induce seroprotection. Statistical analyses confirmed that the influenza vaccine intervention was significantly associated with seroprotection at 1 month, 4 months, and 6 months post-influenza vaccination along with a seroprotection potential approximately more than 3 times higher than in the non-vaccinated groups (RR > 3). Similarly, Kaplan-Meier analysis for the seroprotection (Fig 2) showed a significant difference in the maintenance of the seroprotection for 6 months between those who received the influenza vaccine and those who did not. This result was also corroborated by the marked elevation of antibody anti-influenza titer post-vaccination (Table 9). Apparently that even though 6 months after vaccination the antibody titer had declined to a level that was no longer significantly different with that of the pre-vaccination, the titer was still adequate to yield an effective protection for the subjects. A similar scenario was observed at the 1 month time point; a seroconversion ability of 43% is above the accepted standard of > 30% for successful influenza vaccines for the elderly.

**Table 9:** Geometric Mean Titers (GMT) of the antibody anti-influenza over months.

GMT values	Influenza Vaccine			Probiotics		
	Vaccine (n = 277)	Non-Vaccine (n = 277)	P value	Probiotics (n = 281)	No Probiotics (n = 273)	P value
Month 0 (pre-vaccination)	24.7826	25.4143	0.700	26.4467	23.8151	0.900
Month 1 post-vaccination	297.0046	27.2839	<0.001	97.7981	82.8590	0.800
Month 4 post-vaccination	130.5249	25.8472	0.008	40.9193	54.2132	0.150
Month 6 post-vaccination	99.1869	24.2842	0.500	56.1377	42.9065	0.400

The trivalent influenza vaccine included A/California/7/2009(H1N1)pdm09-like virus, A/Texas/50/2012(H3N2)-like virus, dan B/Massachusetts/2/2012-like virus strains.

However, there was no significant difference in ILI incidence between the vaccinated and control groups, with a p value = 1.000, a relative ILI incidence risk (RR) of 1.0, and a null Absolute Risk Reduction (ARR) factor. The fact that very few participants experienced ILI during the course of the study precludes any strong conclusions about the true effectiveness of this vaccine against influenza infections. Indeed, statistical analyses that had been conducted to find the relationship between vaccines and ILI, have shown that the influenza vaccines did not provide a significant level of protection against ILI, with a RR = 1, which means that participants in both the vaccine and placebo groups have a relatively similar risk of developing ILI. This could be attributable to our sampling strategy. As our study population was recruited from people attending to health education activities in various Primary Health Care Center (Puskesmas) of the East Jakarta district, it is likely that the uptake of lifestyle- and hygiene-related ILI prevention measures provided through the health education activities was successful in this specific population, thereby reducing the overall ILI incidence in our study sample.

With regards to the probiotics intervention, there was a tendency towards a higher seroconversion status at the 1- and 4-month time points in the vaccine + probiotics supplementation group compared to the vaccine + placebo group, suggesting a potential role for probiotics role in enhancing seroconversion. As our study appeared to have been underpowered to detect ILI incidence in this particular segment of the population, this trend warrants further studies to establish the effect of probiotics supplementation on relative ILI risk in the elderly.

## Conclusions

The tested influenza vaccines significantly induced seroprotection and seroconversion in an elderly population. However, as the overall relative risk of ILI events was low in our population, no reduction in the relative risk of ILI events was observed in vaccinated individuals. While probiotic supplementation did not influence seroprotection and seroconversion in our study population, the observed trend towards a reduction in ILI incidence warrants for further assessments in a larger, at-risk population.

## Supporting information

S1 File This is the anonymised raw data of antibody titre of the study subjects. (PDF)

S2 File This is the study protocol in original version (in Bahasa Indonesia). (PDF)

S3 File This is the study protocol in translated version (in English). (PDF)

S1 Checklist This is the consort checklist of the manuscript. (DOC)
