# Supplementary material for: A randomized controlled trial to evaluate the effect of influenza vaccination and probiotic supplementation on immune response and incidence of influenza-like illness in an elderly population in Indonesia
Source: PLoS One. 2021 Dec 16;16(12):e0250234. doi: 10.1371/journal.pone.0250234 (PMC8675694; doi:10.1371/journal.pone.0250234)
Supplement: S1 File — (PDF) [file pone.0250234.s001.pdf]

	Pre-Vaccine			Month 1			Month 4			Month 6		
No	A/California	B/Massachusetts	A/Texas	A/California	B/Massachusetts	A/Texas	A/California	B/Massachusetts	A/Texas	A/California	B/Massachusetts	A/Texas
1	20	20	80	1280	160	320	160	80	160	80	40	80
2	80	20	640	80	20	640	80	20	640	40	20	640
3	10	10	20	1280	640	640	640	320	320	160	160	320
4	80	20	320	640	320	1280	320	80	1280	160	40	1280
5	10	40	160	1280	80	160	320	80	160	320	80	160
6	20	20	640	160	40	640	80	40	320	80	20	160
7	10	10	160	80	320	160	40	160	160	40	80	160
8	10	20	40	80	160	320	40	40	160	20	40	80
9	10	10	160	80	640	320	40	160	160	20	80	160
10	20	20	160	2560	320	320	320	160	320	320	80	320
11	10	10	20	1280	20	320	640	40	160	640	40	160
12	40	20	160	320	40	1280	320	80	1280	320	80	640
13	20	40	80	640	640	640	160	320	160	160	320	160
14	20	40	20	160	640	160	40	160	80	40	160	40
15	40	160	640	640	320	1280	320	320	640	160	160	640
16	20	10	160	320	40	320	160	20	80	160	20	80
17	10	160	40	320	320	160	160	320	160	160	320	80
18	10	40	40	80	320	320	40	320	160	40	320	160
19	20	40	40	80	1280	2560	40	320	640	40	320	640
20	80	20	40	320	160	320	80	80	160	80	80	160
21	20	80	80	10	10	40	40	640	160	2560	320	80
22	20	40	80	160	640	640	80	160	320	40	160	160
23	20	20	320	80	1280	1280	320	640	1280	160	320	1280
24	10	320	80	80	640	80	20	320	80	10	160	40
25	160	320	160	640	320	640	320	320	320	320	320	320
26	20	40	80	640	320	2560	160	160	1280	160	80	640
27	40	80	160	640	640	1280	320	320	640	160	160	320
28	20	40	20	80	640	40	40	160	20	40	160	20
29	10	40	20	40	320	640	10	160	320	20	160	320
30	20	20	20	640	160	40	80	40	20	80	40	10
31	10	40	20	2560	1280	160	640	640	80	640	320	40

32	20	40	320	160	640	640	40	320	640	40	320	320
33	320	20	10	1280	640	1280	640	320	640	320	160	320
34	20	80	160	640	160	320	160	160	160	80	80	80
35	160	80	640	640	160	1280	640	160	1280	640	80	640
36	160	40	40	1280	640	1280	640	320	320	320	320	320
37	160	160	320	640	2560	1280	320	1280	640	320	640	640
38	20	80	160	40	320	640	10	160	160	10	160	160
39	10	20	10	40	320	160	10	160	80	10	160	40
40	40	20	80	2560	640	640	640	160	640	320	160	640
41	80	640	160	160	320	1280	80	160	1280	80	160	1280
42	40	40	40	320	40	160	320	40	20	160	40	20
43	10	320	640	640	640	640	320	640	320	160	320	160
44	10	40	160	80	2560	2560	80	2560	2560	40	2560	2560
45	40	20	80	640	320	640	640	160	320	640	160	640
46	10	40	10	80	80	160	80	80	160	40	80	80
47	10	40	20	320	640	320	320	640	320	160	320	160
48	40	640	160	1280	320	160	320	160	80	160	160	80
49	20	40	40	640	320	160	640	320	80	320	160	40
50	20	40	40	640	320	1280	320	80	320	320	40	160
51	10	40	20	640	80	80	80	80	40	80	80	20
52	40	80	80	80	640	1280	20	320	640	20	320	640
53	10	160	160	40	160	1280	20	160	1280	20	160	640
54	20	10	10	160	40	160	40	40	160	40	40	160
55	40	80	40	320	1280	1280	80	320	160	80	160	80
56	10	20	20	10	80	160	10	40	40	640	40	20
57	320	20	10	320	320	40	80	320	20	80	160	10
58	10	20	80	640	80	80	160	40	40	80	40	40
59	20	40	640	640	160	640	640	80	320	320	40	320
60	10	20	20	160	640	1280	80	160	160	40	80	80
61	40	40	20	1280	80	2560	640	80	640	320	40	320
62	640	20	40	640	80	640	160	40	640	80	40	640
63	20	80	320	2560	320	320	640	160	160	320	160	160
64	80	160	320	1280	640	2560	320	320	1280	320	320	80
65	10	20	320	1280	320	1280	640	160	1280	320	160	1280
66	40	40	80	2560	40	80	1280	40	40	1280	40	20

67	20	40	320	20	160	320	10	80	320	20	80	320
68	80	160	640	320	80	1280	160	80	640	80	80	640
69	20	40	80	1280	640	1280	640	320	1280	640	320	1280
70	40	20	20	640	640	160	320	320	80	320	160	80
71	10	20	160	320	80	160	160	80	160	80	40	160
72	20	20	320	1280	20	1280	320	20	1280	160	20	640
73	20	40	20	80	640	1280	20	320	320	20	320	160
74	20	20	160	640	1280	640	640	640	640	640	640	320
75	40	80	160	640	320	640	160	40	640	80	80	640
76	40	80	80	20	20	80	80	160	320	20	80	160
77	80	640	160	80	640	160	40	640	80	40	640	80
78	40	40	80	640	320	320	20	40	40	80	160	160
79	40	640	160	640	640	320	320	640	160	320	640	160
80	80	80	160	640	80	1280	160	80	640	160	80	320
81	80	160	160	1280	640	320	640	160	160	80	1280	320
82	40	160	160	80	320	320	80	160	640	20	160	320
83	160	40	160	640	640	640	640	320	640	640	160	640
84	20	160	40	640	320	80	160	160	40	160	80	640
85	20	40	320	40	640	1280	10	320	640	20	320	640
86	40	160	80	1280	1280	320	160	640	160	80	320	80
87	20	320	80	1280	640	640	320	640	320	160	320	160
88	40	80	320	1280	320	640	640	80	320	640	80	320
89	20	40	20	640	320	80	320	80	40	160	40	20
90	40	640	160	160	1280	640	80	1280	320	40	1280	160
91	10	40	20	1280	160	640	640	160	320	320	80	160
92	10	10	20	80	320	320	20	80	160	10	40	160
93	40	80	320	160	320	640	40	320	320	40	320	320
94	10	20	40	80	320	160	20	80	80	20	80	40
95	20	40	40	160	320	1280	40	320	640	40	160	640
96	160	40	160	1280	5120	1280	640	2560	1280	320	1280	1280
97	40	40	80	80	80	160	80	80	160	40	40	160
98	20	80	40	40	640	320	40	1280	160	40	1280	320
99	10	320	80	640	1280	1280	320	320	1280	160	320	640
100	80	20	40	80	640	1280	40	640	320	20	320	160
101	160	80	320	640	1280	1280	640	1280	1280	640	640	640

102	40	40	40	1280	80	2560	320	80	1280	320	80	1280
103	40	40	80	160	80	320	80	40	160	160	40	160
104	20	80	80	320	1280	640	160	640	160	80	320	80
105	40	80	40	640	160	160	640	160	160	320	160	160
106	40	20	40	160	160	1280	160	80	1280	80	80	1280
107	40	160	160	160	320	80	160	320	80	80	160	20
108	80	80	80	640	1280	80	640	640	80	640	640	40
109	40	40	40	160	320	1280	80	160	640	40	160	320
110	40	40	320	160	320	1280	160	80	640	80	80	640
111	10	10	40	320	80	640	80	40	640	320	640	1280
112	10	20	10	40	80	80	40	80	160	20	40	80
113	40	40	80	160	1280	1280	80	640	640	40	320	320
114	80	320	40	640	320	5120	160	320	2560	80	160	1280
115	10	20	20	320	80	640	320	80	640	320	80	320
116	10	320	10	2560	160	20	1280	320	20	640	80	20
117	10	10	10	640	80	40	80	40	20	40	40	20
118	20	160	80	160	320	320	80	160	160	80	160	160
119	40	20	40	5120	640	1280	2560	320	320	2560	320	320
120	20	20	10	2560	640	640	640	80	160	320	80	160
121	20	320	20	640	320	320	160	320	80	160	320	40
122	80	20	40	1280	80	160	640	80	160	640	20	160
123	20	20	80	640	40	640	20	20	40	640	40	320
124	20	20	20	640	640	2560	320	640	2560	320	320	2560
125	40	40	40	2560	320	1280	640	80	160	1280	80	160
126	40	80	40	2560	640	1280	1280	320	320	640	160	160
127	20	40	80	640	1280	640	160	320	320	160	320	160
128	80	160	80	320	640	320	80	160	80	80	160	80
129	20	20	20	640	160	160	320	80	80	160	80	80
130	40	160	40	320	320	160	160	160	80	80	160	80
131	20	160	40	2560	160	640	320	160	160	160	160	80
132	10	20	40	640	40	160	320	40	80	320	20	40
133	20	320	40	160	640	1280	40	320	640	20	320	640
134	10	160	80	80	160	640	80	160	320	40	160	160
135	10	320	160	20	1280	1280	10	640	1280	10	320	1280
136	40	160	80	160	320	160	80	160	160	80	160	80

137	80	320	20	160	320	40	160	320	20	80	320	20
138	20	20	80	320	160	320	80	80	160	80	80	160
139	20	640	40	1280	640	320	640	640	160	40	160	160
140	10	40	10	10	10	40	10	40	20	10	40	20

Influenza Vaccine and Probiotic Placebo

		Pre-Vaccine			Month 1			Month 4			Month 6	
No	A/California	B/Massachusetts	A/Texas	A/California	B/Massachusetts	A/Texas	A/California	B/Massachusetts	A/Texas	A/California	B/Massachusetts	A/Texas
1	10	160	40	160	640	320	80	320	160	80	320	160
2	20	20	80	20	20	80	20	20	80	20	20	80
3	10	10	40	40	80	1280	20	40	320	10	20	320
4	20	320	80	640	80	640	640	320	160	320	320	80
5	10	40	320	320	80	640	80	40	320	40	40	320
6	10	10	20	640	20	80	160	20	80	160	20	40
7	10	10	20	640	160	80	80	80	40	80	40	40
8	10	20	320	80	10	640	20	10	320	20	10	320
9	10	20	40	80	160	80	40	80	40	40	40	40
10	20	320	320	320	320	320	80	10	160	80	160	160
11	40	160	160	80	1280	640	80	640	320	80	640	320
12	10	1280	80	80	2560	2560	40	1280	1280	80	2560	1280
13	10	20	80	40	160	1280	20	80	640	20	80	640
14	10	20	20	320	40	20	160	40	10	80	40	20
15	20	160	160	320	160	320	160	160	320	80	160	320
16	20	40	80	160	40	320	160	40	160	80	20	160
17	10	20	40	40	40	2560	80	20	1280	40	40	1280
18	80	40	40	320	160	320	320	80	160	320	40	80
19	20	40	160	80	160	1280	80	80	640	80	80	640
20	80	40	20	2560	160	1280	2560	160	640	1280	80	640
21	10	40	40	160	2560	160	80	1280	160	80	1280	80
22	40	20	320	640	160	1280	640	160	1280	640	160	1280
23	20	80	640	2560	160	640	1280	160	320	1280	80	320

24	40	20	20	320	320	640	160	160	320	160	160	320
25	10	20	40	320	80	320	20	20	80	40	40	80
26	20	20	160	160	1280	1280	80	640	1280	40	640	1280
27	20	320	80	640	80	640	640	320	160	320	320	80
28	40	20	40	1280	320	1280	320	40	640	160	40	320
29	10	20	20	160	160	320	20	80	80	20	80	80
30	20	40	160	640	2560	1280	160	1280	1280	80	640	640
31	10	40	40	640	80	1280	160	80	1280	160	80	1280
32	40	160	160	320	320	1280	20	80	320	20	40	160
33	10	160	20	80	320	1280	40	160	1280	20	160	1280
34	10	40	160	80	2560	2560	80	2560	2560	40	2560	2560
35	10	80	40	1280	640	1280	640	160	640	320	160	640
36	20	320	80	1280	640	1280	1280	320	1280	1280	160	640
37	20	40	160	1280	160	1280	80	80	640	160	160	640
38	10	40	80	80	640	640	40	320	320	20	320	320
39	10	40	160	640	1280	1280	160	640	640	80	320	640
40	20	40	20	320	320	640	160	320	160	160	160	160
41	40	40	80	640	160	160	320	40	80	160	40	80
42	10	80	320	320	1280	1280	160	640	1280	40	320	640
43	10	10	20	160	160	1280	80	80	640	80	80	320
44	10	10	20	40	80	640	20	80	160	20	80	160
45	10	10	40	640	160	160	160	80	80	80	40	80
46	10	10	10	160	320	640	40	80	160	40	80	80
47	10	160	160	320	640	320	160	640	160	160	640	80
48	10	40	160	640	320	1280	320	320	1280	160	160	1280
49	40	40	640	80	80	640	40	80	320	20	40	160
50	80	20	1280	320	1280	1280	160	1280	640	80	1280	640
51	20	20	40	320	320	1280	80	160	640	40	80	320
52	80	10	80	640	20	320	320	20	160	160	20	160
53	40	80	80	2560	640	640	640	160	160	320	160	80
54	10	20	640	40	40	640	40	40	640	20	40	640
55	20	160	40	640	640	2560	160	640	2560	160	640	1280
56	160	320	640	320	320	640	160	160	320	80	160	160
57	10	640	80	1280	1280	640	640	1280	640	640	1280	640
58	10	20	40	1280	40	320	640	40	320	320	20	320

59	80	20	80	640	80	320	80	40	80	40	40	80
60	20	40	20	20	80	320	20	80	80	10	40	40
61	20	10	20	160	320	640	40	160	160	40	160	160
62	10	20	640	640	160	640	320	80	320	160	80	160
63	10	10	40	320	40	1280	80	20	1280	80	40	1280
64	10	160	160	40	640	320	20	640	320	20	640	160
65	10	640	10	320	640	1280	80	320	160	40	320	80
66	20	160	320	160	1280	640	40	640	320	40	640	320
67	80	80	80	1280	320	320	160	80	80	160	80	40
68	20	40	40	640	160	80	160	80	40	160	80	40
69	20	20	40	320	320	640	320	320	640	320	160	640
70	20	80	80	40	320	2560	80	160	1280	40	160	640
71	40	80	80	80	80	1280	20	80	320	20	40	160
72	20	80	320	320	320	320	40	160	160	40	160	160
73	80	80	320	1280	320	1280	640	160	1280	640	320	1280
74	160	160	320	640	1280	1280	80	640	160	160	640	640
75	20	80	640	320	640	640	40	160	640	40	160	320
76	40	640	80	160	1280	1280	40	640	320	20	640	320
77	20	320	160	1280	320	320	640	640	320	640	320	160
78	80	80	320	320	80	320	320	80	320	160	20	160
79	80	160	160	80	1280	1280	20	1280	1280	10	640	640
80	40	320	320	640	320	640	320	80	320	160	80	160
81	40	80	160	160	160	1280	80	160	640	20	80	640
82	80	160	80	160	160	40	80	160	40	40	80	20
83	80	320	640	320	1280	1280	320	640	640	80	640	640
84	40	40	80	640	640	320	320	640	320	160	320	160
85	40	40	80	640	160	320	160	80	160	80	40	80
86	20	40	80	160	10	2560	40	40	1280	20	40	1280
87	40	80	160	80	640	2560	80	320	1280	40	160	1280
88	20	640	160	2560	1280	1280	1280	640	1280	1280	640	1280
89	20	40	40	80	320	1280	80	160	640	80	160	640
90	40	80	640	640	640	1280	320	320	640	160	160	640
91	40	20	40	40	40	320	80	40	320	40	20	160
92	40	80	80	1280	160	320	320	160	160	80	80	40
93	40	320	160	320	640	640	320	640	640	320	640	320

94	20	160	640	80	1280	1280	80	1280	1280	40	640	640
95	40	40	80	640	640	640	320	160	320	160	160	160
96	40	160	160	2560	2560	640	40	80	320	640	1280	160
97	20	20	160	40	160	1280	40	40	640	40	40	640
98	20	20	640	160	160	320	80	80	320	80	80	320
99	20	80	20	320	160	40	80	160	40	80	160	40
100	160	640	80	320	1280	1280	160	640	1280	160	640	1280
101	40	80	40	640	1280	1280	160	320	1280	160	320	640
102	20	40	40	320	1280	2560	80	640	1280	80	640	640
103	10	40	160	20	640	160	40	640	160	20	160	80
104	20	80	20	320	160	640	160	80	320	160	80	160
105	20	20	40	80	40	160	40	20	160	40	20	160
106	160	80	80	640	640	1280	320	640	1280	320	320	640
107	10	20	20	40	640	320	20	320	160	10	160	160
108	80	20	40	80	320	80	80	160	80	40	80	80
109	40	40	40	160	80	320	160	80	160	160	80	160
110	20	80	40	80	160	320	40	160	160	40	80	160
111	10	80	80	80	160	320	20	80	160	20	80	160
112	20	20	320	160	160	640	40	80	640	40	40	320
113	80	160	40	80	160	40	40	80	40	80	80	40
114	40	320	80	160	320	2560	40	160	640	40	160	640
115	10	20	40	≥ 640	640	2560	320	640	1280	160	160	640
116	10	10	160	160	160	1280	80	80	1280	20	40	1280
117	10	10	40	40	160	640	40	160	320	40	160	320
118	10	20	80	80	320	640	40	80	640	40	160	1280
119	160	160	40	1280	160	320	640	160	160	640	80	160
120	10	10	640	5120	160	1280	2560	160	1280	1280	80	640
121	20	10	10	2560	320	10	1280	160	10	640	160	20
122	20	80	80	320	1280	1280	80	640	320	80	320	160
123	20	320	80	1280	640	80	640	640	80	640	640	80
124	40	160	40	80	160	160	80	160	80	80	160	80
125	40	80	80	2560	640	1280	640	640	640	320	640	640
126	20	1280	640	160	1280	640	160	640	640	40	320	320
127	10	20	20	320	40	80	80	40	40	20	10	20
128	20	160	40	320	160	640	40	160	320	640	320	160

129	10	160	20	1280	1280	1280	320	320	640	160	320	640
130	40	80	80	10	640	1280	320	320	1280	160	320	1280
131	10	10	20	2560	1280	160	320	320	40	160	160	40
132	10	40	10	80	320	80	160	160	40	80	80	20

Probiotic and Vaccine Placebo

	Pre-Vaccine			Month 1			Month 4			Month 6		
No	A/California	B/Massachusetts	A/Texas	A/California	B/Massachusetts	A/Texas	A/California	B/Massachusetts	A/Texas	A/California	B/Massachusetts	A/Texas
1	20	40	40	40	40	80	20	1280	40	20	640	40
2	20	80	40	40	80	80	20	40	40	20	40	40
3	20	40	160	20	160	160	20	80	80	10	80	80
4	10	10	320	20	10	320	20	10	320	20	20	160
5	20	20	160	20	20	160	10	10	80	10	10	80
6	10	80	20	10	80	10	10	40	20	10	40	10
7	40	20	160	40	20	160	20	10	40	40	20	40
8	10	20	40	20	40	40	20	20	40	20	40	40
9	10	20	640	10	10	80	10	20	80	10	10	80
10	40	40	160	80	80	80	40	20	80	40	20	80
11	20	40	20	20	40	20	20	40	40	20	40	20
12	20	40	20	20	80	20	40	40	20	40	80	20
13	10	40	40	10	20	40	10	20	40	20	40	40
14	20	40	40	20	20	20	20	80	40	80	2560	80
15	160	10	20	160	20	20	80	20	20	80	20	40
16	20	20	80	20	20	80	20	20	80	20	20	160
17	20	5120	20	10	5120	20	10	2560	40	10	2560	20
18	20	20	80	20	20	80	20	10	40	20	20	40
19	20	40	40	40	80	40	40	40	20	40	40	40
20	20	80	320	20	80	320	20	80	160	20	40	320
21	320	10	80	160	10	80	160	10	80	160	20	80
22	80	160	80	80	160	80	40	160	80	40	160	80
23	20	10	10	20	10	10	10	10	10	10	10	10

24	10	10	10	10	10	20	10	10	10	10	10	20
25	20	80	10	20	80	20	10	80	<10	10	80	10
26	40	640	320	40	640	320	40	320	160	40	320	160
27	10	10	20	10	10	40	10	10	20	10	10	40
28	10	40	20	10	40	10	10	20	10	10	20	20
29	10	40	320	10	40	160	10	40	80	10	40	160
30	20	320	320	20	320	320	40	320	320	10	320	160
31	160	1280	40	160	1280	80	160	640	80	160	640	80
32	20	20	160	20	40	160	20	20	160	20	40	160
33	10	20	20	20	40	40	10	20	40	20	40	40
34	80	40	80	80	20	40	40	20	40	40	20	20
35	20	40	40	20	80	40	40	40	40	40	40	40
36	20	160	160	20	160	80	10	160	80	80	160	320
37	10	40	320	20	40	320	20	40	160	10	20	160
38	40	20	40	40	80	80	80	80	160	20	20	40
39	10	20	40	10	40	80	10	40	80	10	40	80
40	10	20	80	10	10	80	80	80	160	10	10	40
41	40	20	10	40	20	10	80	40	10	80	20	20
42	10	20	160	10	20	80	10	20	80	10	20	80
43	20	40	40	20	40	40	20	40	40	20	40	40
44	640	160	80	1280	80	80	1280	160	80	1280	160	80
45	20	160	10	10	160	10	20	160	10	20	160	10
46	20	20	20	10	10	10	10	10	10	10	10	10
47	10	20	20	10	40	20	10	20	20	10	20	20
48	10	80	80	10	80	80	10	160	80	10	80	80
49	20	40	640	10	40	640	10	40	640	10	40	320
50	10	20	320	10	20	320	10	20	320	10	20	320
51	20	40	80	10	20	40	20	20	40	10	10	40
52	10	20	80	10	10	40	10	10	20	10	10	20
53	10	80	320	10	320	320	10	80	320	10	80	320
54	160	40	20	160	40	20	80	80	20	80	40	20
55	10	20	40	10	10	40	10	20	40	10	20	40
56	20	40	160	20	40	160	20	40	160	20	40	160
57	20	40	160	10	20	80	20	40	160	10	20	80
58	40	320	20	40	160	10	40	160	10	40	160	10

59	40	160	40	80	160	40	80	160	40	40	80	40
60	40	160	1280	40	160	640	40	160	640	40	80	640
61	640	40	160	640	20	160	640	40	160	640	20	160
62	80	80	20	160	80	20	80	80	20	80	80	20
63	<10	<10	20	<10	<10	40	<10	10	160	<10	10	160
64	10	80	320	10	40	320	10	40	320	10	40	320
65	40	640	80	40	320	80	10	160	40	20	160	40
66	80	160	40	80	160	40	80	160	40	80	160	40
67	20	160	20	20	160	160	10	80	20	20	80	20
68	40	320	40	40	160	20	40	160	40	40	160	20
69	40	40	80	40	40	40	40	40	40	20	20	20
70	40	20	80	20	10	40	20	10	40	40	10	40
71	20	320	80	20	160	80	20	160	80	20	160	80
72	80	80	160	40	40	80	40	40	80	40	40	80
73	80	160	160	10	20	20	10	10	20	10	20	20
74	20	40	160	20	40	160	20	40	160	20	40	160
75	40	640	40	20	320	20	40	320	40	40	320	20
76	40	320	160	80	320	160	20	320	40	20	320	40
77	80	40	320	40	40	160	80	80	160	40	40	160
78	40	160	80	20	320	40	20	160	40	20	160	20
79	80	80	160	20	40	20	20	40	20	20	20	20
80	400	20	40	40	40	40	80	80	80	40	20	40
81	40	80	80	20	80	80	20	160	80	160	80	80
82	20	40	80	20	40	80	20	40	40	20	40	40
83	160	160	320	80	80	80	80	40	80	80	80	80
84	320	80	640	160	20	320	160	20	320	160	20	160
85	10	40	160	1280	80	160	320	80	160	320	80	160
86	10	10	20	1280	20	320	640	40	160	640	40	160
87	20	640	1280	20	640	1280	10	640	1280	10	320	640
88	40	320	80	1280	1280	1280	640	320	1280	640	320	640
89	20	40	40	20	40	40	10	20	20	10	20	40
90	80	160	80	20	40	40	10	40	20	10	20	20
91	20	160	40	10	160	20	20	160	40	10	160	20
92	20	320	40	40	640	40	10	320	20	640	320	80
93	40	160	80	40	160	80	20	80	20	20	80	20

94	10	20	160	20	40	320	20	160	160	20	80	160
95	20	20	20	20	20	20	20	20	20	20	20	20
96	20	40	80	10	40	80	40	80	80	20	40	80
97	20	40	20	20	40	20	80	80	80	20	80	20
98	10	10	10	10	10	10	10	10	10	160	10	10
99	20	80	40	80	80	80	40	80	160	40	40	80
100	20	40	40	20	40	20	20	40	20	20	20	20
101	40	80	40	20	40	40	20	20	40	20	20	40
102	20	20	160	20	20	160	20	20	160	20	20	160
103	40	20	160	40	10	160	40	10	80	20	20	80
104	20	160	40	20	80	40	40	80	40	20	160	40
105	20	160	160	20	160	80	20	160	80	20	160	40
106	20	20	160	20	320	160	20	160	80	40	160	80
107	10	40	10	10	40	10	10	40	10	10	40	10
108	20	40	80	40	80	80	40	80	80	20	80	40
109	20	160	80	40	160	40	80	160	80	40	160	80
110	20	20	640	160	160	320	80	80	320	80	80	320
111	40	80	40	20	40	40	40	80	160	40	80	160
112	20	40	320	40	40	320	40	40	320	80	80	160
113	10	40	40	80	320	320	40	320	160	40	320	160
114	40	320	40	40	640	40	20	640	20	20	640	20
115	10	160	40	10	320	40	10	160	40	10	80	40
116	10	10	10	40	20	40	40	20	20	40	20	20
117	10	20	10	10	20	10	10	20	20	40	20	10
118	10	10	20	10	10	20	10	10	20	10	10	20
119	20	20	640	20	20	640	40	40	320	20	20	320
120	20	20	80	640	40	640	20	20	40	640	40	320
121	10	40	80	10	40	80	10	40	80	10	40	80
122	40	40	40	20	40	40	40	40	40	40	160	640
123	20	80	40	20	80	40	20	80	40	20	80	40
124	40	20	20	40	20	20	80	20	20	80	20	640
125	40	80	160	40	40	160	40	40	160	20	40	160
126	10	10	10	10	10	20	10	10	20	10	10	20
127	20	40	80	20	80	80	20	80	80	20	40	80
128	10	20	40	10	40	40	10	20	40	20	20	40

129	20	640	320	20	320	320	20	80	160	20	40	80
130	20	20	80	20	40	160	10	20	160	10	20	80
131	40	40	320	40	40	320	40	20	160	40	80	160
132	20	20	20	20	20	20	20	20	20	20	20	20
133	40	40	80	640	160	320	160	80	160	80	40	80
134	20	160	40	20	160	20	160	160	20	160	1280	80
135	320	160	40	320	80	40	160	80	20	160	80	20
136	20	80	40	20	80	40	20	80	40	10	80	20
137	160	320	160	10	10	10	10	10	10	10	10	10

Vaccine Placebo and Probiotic Placebo

		Pre-Vaccine			Month 1			Month 4			Month 6	
No	A/California	B/Massachusetts	A/Texas	A/California	B/Massachusetts	A/Texas	A/California	B/Massachusetts	A/Texas	A/California	B/Massachusetts	A/Texas
1	20	20	80	20	20	80	20	10	80	20	20	80
2	40	20	40	40	20	40	40	20	40	40	10	20
3	20	160	20	20	160	20	20	80	20	20	80	20
4	20	40	40	20	40	40	20	40	40	20	40	40
5	40	80	160	40	80	160	20	80	160	20	80	160
6	20	20	40	20	20	40	20	20	40	20	20	40
7	10	20	10	10	20	10	10	20	10	10	20	10
8	10	10	10	10	10	10	10	10	20	10	10	20
9	10	320	20	10	160	10	10	160	20	10	160	20
10	40	20	80	20	20	80	20	20	80	20	20	80
11	10	20	40	20	40	20	20	20	20	10	20	20
12	10	20	80	20	20	80	20	20	80	20	20	80
13	20	320	80	20	320	40	20	320	80	20	320	80
14	10	20	20	20	20	40	20	20	20	20	20	40
15	80	40	160	80	40	160	160	40	160	80	40	160
16	20	40	320	20	40	320	20	40	160	20	40	320
17	20	40	80	40	160	80	20	20	40	20	20	40
18	80	160	160	20	20	20	20	20	20	20	20	20

19	10	640	40	20	640	80	10	640	80	10	640	80
20	10	20	320	10	20	160	10	20	160	10	20	160
21	10	10	160	20	20	160	20	20	160	20	20	160
22	20	20	80	20	20	40	40	20	40	20	20	40
23	10	80	10	10	40	10	< 10	40	10	10	40	20
24	40	40	20	80	80	80	10	20	10	10	20	10
25	10	20	40	10	20	40	10	10	40	10	10	40
26	20	20	40	20	10	40	10	10	20	10	10	40
27	40	160	320	40	160	320	20	1280	320	20	640	160
28	10	20	80	20	20	80	10	20	40	10	20	80
29	40	40	80	80	80	160	40	40	80	40	40	80
30	10	20	20	10	20	20	10	20	20	< 10	10	20
31	20	80	80	20	80	80	10	80	80	10	40	80
32	10	160	160	< 10	80	80	10	40	80	10	40	80
33	20	20	20	20	20	10	20	20	10	20	20	10
34	10	160	10	10	160	10	20	160	40	10	80	10
35	< 10	320	40	< 10	640	80	< 10	320	40	< 10	320	40
36	10	40	160	20	80	320	20	80	320	20	80	160
37	20	160	80	20	320	80	20	320	80	20	160	80
38	20	40	160	20	20	160	20	40	160	20	40	40
39	320	640	640	320	640	640	320	640	640	40	40	40
40	20	640	640	20	640	640	10	640	320	10	320	160
41	10	20	20	20	20	20	40	80	80	10	20	20
42	10	20	80	10	20	80	10	20	80	10	20	80
43	10	20	20	10	20	10	10	20	10	20	40	20
44	20	40	80	10	20	80	10	160	80	10	160	80
45	20	40	160	40	80	160	20	40	160	10	20	80
46	10	80	40	20	20	80	10	10	40	10	20	80
47	40	160	40	20	160	20	20	160	20	20	160	20
48	10	20	20	< 10	10	20	640	20	20	160	10	10
49	40	160	40	40	80	20	40	80	20	40	80	40
50	10	20	20	10	10	20	10	10	20	10	20	20
51	40	320	160	40	320	160	40	160	160	20	160	80
52	20	160	20	20	160	10	20	160	10	20	80	10
53	40	20	640	40	20	320	40	20	160	20	10	160

54	20	80	80	10	80	80	10	40	40	10	40	80
55	40	10	160	80	10	160	80	10	160	40	10	160
56	40	40	160	40	20	80	20	20	80	20	20	80
57	10	20	80	20	20	160	20	20	80	20	40	80
58	40	160	40	20	80	20	20	80	20	20	80	20
59	40	640	160	20	320	160	20	320	80	20	320	80
60	40	160	80	20	160	40	20	160	20	20	160	40
61	20	160	20	20	160	160	10	80	20	20	80	20
62	40	40	80	40	40	80	40	40	80	40	40	80
63	20	20	20	20	20	20	20	20	20	20	20	20
64	10	40	320	10	40	160	20	40	160	10	40	160
65	20	20	40	10	10	20	10	10	40	10	20	20
66	20	20	20	10	20	20	10	10	20	20	20	20
67	10	20	80	20	20	160	20	20	80	20	40	80
68	10	20	20	10	20	20	10	20	20	20	20	20
69	160	320	640	160	320	640	160	320	640	80	320	640
70	80	160	160	10	40	20	20	40	20	10	40	40
71	80	80	160	160	160	640	40	20	80	20	20	80
72	40	40	80	20	20	20	20	20	40	10	20	20
73	160	640	160	40	640	40	640	320	40	640	320	20
74	20	80	320	20	80	320	40	80	160	20	40	160
75	40	160	160	20	80	20	40	160	80	20	80	20
76	40	80	320	20	40	160	10	40	320	10	20	160
77	20	80	640	20	80	640	20	80	640	40	80	640
78	80	80	80	40	80	80	40	80	80	10	20	40
79	40	80	80	20	160	80	320	160	160	80	80	80
80	80	80	80	40	40	80	40	80	80	40	80	80
81	40	80	80	20	80	80	40	40	80	10	20	40
82	40	80	320	10	80	80	10	80	160	10	40	160
83	20	160	160	40	160	160	10	40	80	10	40	80
84	10	320	20	20	640	40	10	640	20	10	640	20
85	20	160	20	40	640	80	20	160	20	40	160	80
86	160	160	320	160	160	320	160	80	320	80	80	320
87	20	80	40	20	40	20	20	40	20	40	40	40
88	20	40	160	40	40	80	20	20	80	20	20	80

89	40	80	40	40	40	20	40	80	40	20	40	40
90	20	20	40	40	40	80	40	40	80	40	80	80
91	40	40	40	80	160	160	40	160	160	40	160	160
92	80	40	160	80	80	160	40	40	160	40	40	80
93	20	20	40	20	20	40	20	20	20	20	20	40
94	20	20	20	20	20	40	20	20	40	20	20	40
95	40	40	40	80	80	80	80	80	80	80	40	80
96	20	20	80	40	40	80	20	20	80	20	20	80
97	160	40	20	160	40	40	160	40	40	80	40	40
98	20	640	160	20	320	160	20	160	160	40	160	160
99	160	80	80	80	40	40	80	40	40	40	40	40
100	20	160	40	40	320	80	40	320	80	40	320	80
101	640	160	160	640	160	160	640	160	160	640	160	160
102	20	160	160	20	80	160	20	160	160	20	80	160
103	20	20	20	20	40	40	20	160	20	20	160	20
104	320	160	80	160	80	80	160	160	80	80	80	80
105	20	40	80	20	40	80	20	40	80	10	40	80
106	40	80	80	20	80	40	40	80	80	20	40	20
107	20	40	20	10	20	20	10	40	20	10	10	10
108	< 10	160	10	10	80	10	10	80	10	10	80	10
109	10	40	40	10	20	20	20	40	40	10	40	40
110	10	40	20	10	40	20	10	40	20	10	40	20
111	< 10	10	20	80	80	80	< 10	10	20	10	10	20
112	10	320	10	10	320	20	10	160	10	10	160	10
113	160	40	10	160	40	20	160	40	20	80	40	10
114	80	20	160	80	20	80	80	40	80	40	20	40
115	40	10	20	< 10	<10	<10	10	10	10	10	10	10
116	20	320	80	20	320	80	20	640	160	20	640	160
117	40	160	320	20	80	320	20	80	160	20	80	160
118	20	10	80	10	< 10	80	10	10	40	10	< 10	40
119	20	< 10	20	20	< 10	20	20	< 10	20	20	< 10	20
120	40	10	80	80	10	80	80	10	80	40	20	40
121	10	40	320	10	40	320	10	40	160	10	40	160
122	10	10	10	10	10	10	1280	2560	2560	640	20	10
123	10	160	20	20	320	20	20	80	20	10	80	20

124	10	10	80	10	10	80	10	10	80	10	10	80
125	< 10	10	10	< 10	10	10	10	20	20	80	80	80
126	80	40	160	160	80	640	160	40	320	160	40	320
127	10	20	20	20	160	80	10	160	20	10	160	20
128	10	40	80	10	40	80	10	40	80	< 10	40	40
129	20	80	40	20	80	40	20	80	40	20	40	40
130	20	160	160	10	160	160	10	160	160	10	80	160
131	40	80	160	40	80	160	20	40	80	20	40	80
132	80	40	320	80	40	160	80	40	160	80	40	160
133	20	80	80	40	160	160	40	160	160	10	80	40
134	10	20	20	10	20	20	10	20	40	10	20	20
135	10	10	160	< 10	10	160	10	10	80	10	10	80
