# Supplementary material for: A randomized controlled trial to evaluate the effect of influenza vaccination and probiotic supplementation on immune response and incidence of influenza-like illness in an elderly population in Indonesia
Source: PLoS One. 2021 Dec 16;16(12):e0250234. doi: 10.1371/journal.pone.0250234 (PMC8675694; doi:10.1371/journal.pone.0250234)
Supplement: S2 File — (PDF) [file pone.0250234.s002.pdf]

“Peran Probiotik dan Vaksinasi Influenza dalam Menurunkan Kejadian Influenza-Like Illness (ILI) pada Lansia”

Protokol Penelitian

PENGANTAR

Influenza adalah penyebab utama mortalitas dan morbiditas di seluruh dunia. Memang, virus influenza hanya dapat menyebabkan gejala minimal, tetapi juga dapat menyebabkan komplikasi yang parah dan mematikan. Secara umum, infeksi virus influenza menyebabkan *Acute Respiratory Illness* (ARI). Namun, karena gejala ISPA juga dapat disebabkan oleh agen infeksi lain dan tidak spesifik untuk virus influenza, rangkaian gejala ini disebut sebagai *Influenza-Like Illness* (ILI). Di Indonesia, saat ini tidak ada laporan tentang prevalensi penyakit ini. Berdasarkan gejala yang digunakan untuk mendefinisikan ISPA, prevalensi diperkirakan 25%. Berbagai penelitian telah menunjukkan bahwa virus influenza dan *Respiratory Syncytial Virus* (RSV) sering dikaitkan dengan penyakit pernapasan akut yang memerlukan rawat inap, terutama pada populasi lansia dan pasien dengan kronis sebelumnya. penyakit. Inilah sebabnya mengapa individu berusia 65 tahun atau lebih dianggap sebagai kelompok yang paling rentan, mewakili 90% dari kasus komplikasi terkait influenza yang dilaporkan.

Vaksinasi dianggap sebagai metode pencegahan utama dalam penatalaksanaan influenza. Keampuhan vaksin dalam mencegah penyakit dapat disimpulkan berdasarkan kemanjuran dan keefektifannya dalam menginduksi serokonversi, menganugerahkan perlindungan, dan mengurangi kejadian ILI. Namun, studi klinis tentang efektivitas dan kemanjuran vaksin influenza pada populasi lansia telah menghasilkan hasil yang bertentangan.

Immunosenescence, yang mengacu pada proses penuaan sistem kekebalan tubuh yang dicerminkan oleh peningkatan insiden infeksi pada lansia, telah diusulkan sebagai penyebab yang mendasari berkurangnya respons imunisasi terhadap vaksin yang diamati pada populasi lansia. Diperlukan strategi baru untuk meningkatkan efektivitas vaksin influenza pada lansia, baik dengan meningkatkan respons imun individu atau formulasi vaksin. Dalam penelitian ini, kami mengeksplorasi apakah probiotik dapat meningkatkan respons kekebalan yang dipicu oleh vaksin influenza trivalen di lansia, dan mengurangi insiden ILI pada populasi ini.

Tujuan: Untuk menyelidiki efek vaksinasi influenza dengan atau tanpa suplementasi probiotik pada respon imun dan kejadian penyakit mirip influenza (ILI) pada lansia.

Design Penelitian

Penelitian ini adalah uji coba acak, tersamar ganda, terkontrol plasebo dengan desain faktorial yang membandingkan kemanjuran dua intervensi, vaksin influenza dan probiotik, dalam mengurangi risiko ILI pada lansia. Peserta yang memenuhi syarat secara acak dibuat menjadi empat kelompok intervensi: vaksin influenza + probiotik; vaksin influenza + plasebo; plasebo + probiotik; dan keduanya plasebo. Penelitian ini dilakukan di seluruh Kabupaten Pulo Gadung, Jakarta Timur, antara bulan April dan Desember 2015, yang merupakan periode yang meliputi musim flu.

1.1 Kriteria Seleksi dan Pengunduran

1.1.1 Kriteria Inklusi

1. Seluruh lansia yang datang ke kegiatan vaksinasi dan penyuluhan kesehatan yang diadakan di seluruh wilayah Puskesmas Kecamatan Jakarta Timur, yaitu laki-laki dan perempuan berusia ≥ 60 tahun.
2. Dalam keadaan sehat dalam 6 bulan terakhir, dan pada prosedur awal penelitian.
3. BMI 17.5 - 29.9

4. Status mental dengan mini mental state examination (MMSE) tergolong sehat (skor MMSE 28-30).
5. Mobilitas baik.

1.1.2 Kriteria Eksklusi

1. Memiliki kontraindikasi terhadap pemberian Vaksin Influenza
    a. Alergi berat terhadap telur ayam atau protein ayam
    b. Pernah menderita Sindrom Guillain-Barre
2. Sedang menjalani pengobatan terkait modulasi sistem imun, contohnya:
    a. Terapi immunoglobulin intravena dalam 4 minggu terakhir
    b. Terapi imunosupresan dan atau kortikosteroid setara dengan prednisone ≥ 20 mg/hari, sedang dijalani lebih dari 2 minggu, atau baru dihentikan kurang dari 3 bulan sebelum penelitian.
3. Telah mendapat vaksinasi influenza kurang dari satu tahun sebelumnya.
4. Sedang mengkonsumsi agen probiotik, baik dalam bentuk pabrikan atau alamiah lebih dari 7 hari

1.1.3 Prosedur Rekrutmen

1. Pemeriksaan kelayakan partisipan

    Dengan data jumlah populasi terjangkau di seluruh wilayah Puskesmas Kecamatan Jakarta Timur. Peneliti melakukan pemeriksaan kelayakan partisipan:

    i. Pengisian kuesioner kelayakan.
    ii. Pemeriksaan fisik diperlukan.
2. Penerimaan partisipan

    Setelah partisipan dipilih, mereka dikumpulkan dalam masing-masing posyandu lansia dan diberikan penjelasan mengenai penelitian.
3. Partisipan yang menyetujui untuk ikut serta dalam penelitian menandatangani *informed consent*.

1.1.4 Kriteria Pengunduran atau Drop Out

1. Pasien meninggal.
2. Pasien kehilangan kontak selama pengamatan dengan berbagai macam penyebab, seperti pindah tempat tinggal.

3. Pasien dengan sadar dan/atau tanpa pengaruh menyatakan ingin berhenti dari penelitian.
4. Pasien yang mendapatkan terapi modulasi imun atau imunosupresan selama penelitian oleh sebab apapun.

1.2 Intervensi dan Pemeriksaan Laboratorium

1.2.1 Vaksin Influenza

Vaksinasi influenza menggunakan vaksin merk *Flubio*® dari Biofarma yang merupakan *Trivalent Inactivated Influenza Vaccine (TIV)* mengandung haemaglutinin dari antigen virus influenza, dan telah dibuat dengan strain virus sesuai rekomendasi WHO untuk hemisfir utara (*northern hemisphere*) tahun 2014-2015 (WHO, 2014). Vaksin berupa suspensi jernih yang diberikan untuk injeksi 0,5 ml IM pada setiap subjek penelitian yang terpilih. Setiap dosis mengandung influenza A/California/7/2009 (H1N1), A/Texas/50/2012(H3N2), dan B/Massachusetts/2/2012 dengan dosis hemagglutinin masing-masing 15 mg.

1.2.2 Probiotik

Probiotik yang digunakan dalam penelitian ini adalah 2 milyar cfu organisme yang terdiri dari *Lactobacillus acidophilus* Rossel-52 dan *Lactobacillus rhamnosus* Rosell-11, dengan zat tambahan: maltodextrin 211 mg, magnesium stearat 8 mg, asam askorbat 1 mg. Secara fisik zat pembawa tersebut berwarna putih, dimasukkan dalam kapsul pabrikan berwarna bening dengan ukuran nomor 2, dengan nama Lacidofil® yang diedarkan oleh PT Dexa Medica Probiotik ini diberikan 2 x perhari, selama 6 bulan terus menerus.

1.2.3 Plasebo

Kontrol vaksin yang disuntikkan adalah plasebo NaCl 0,9% 0,5 ml. Kontrol probiotik adalah plasebo probiotik dengan ukuran dan warna kapsul yang sama dengan probiotiknya, tetapi tidak menimbulkan efek terapi. Plasebo probiotik ini berupa zat amilum trisi steril, yang dikemas dalam kapsul bening ukuran nomor 2.

1.2.4 Pemeriksaan Laboratorium

Pemeriksaan Laboratorium membutuhkan bahan dan alat sebagai berikut:

1. Reagen hemaglutinin untuk virus influenza serotipe H1N1, H3N2, dan B sesuai strain yang beredar saat ini sebanyak dua kali jumlah sampel (untuk pengukuran kadar HI serum sebelum dan sesudah vaksinasi).
2. Vaccutainer untuk menyimpan darah dan pengiriman sampel darah ke laboratorium, sebanyak dua kali jumlah sampel.
3. Spuit 3 cc sebanyak tiga kali jumlah sampel untuk mengambil sampel darah sebelum dan sesudah vaksinasi, serta penyuntikan vaksin influenza.
4. Kapas alkohol untuk asepsis.
5. Alat pemeriksaan titer HI Influenza yang sudah dikalibrasi oleh Litbangkes.

1.3 Prosedur Penelitian

1. Pengumpulan data lansia dari Posyandu Lansia yang berada di wilayah Puskesmas Kecamatan Pulo Gadung, Jakarta Timur. Data berasal dari para kader posyandu lansia di bawah Binaan Klinik Dokter Keluarga Kayu Putih yaitu:
   a. Posyandu Lansia Kayu Putih
   b. Posyandu Lansia Pisangan Timur 1
   c. Posyandu Lansia Pisangan Timur 2
   d. Posyandu Rawamangun
   e. Posyandu Cipinang Timur 1
   f. Posyandu Cipinang Timur 2
2. Penyebaran undangan kepada seluruh peserta posyandu lansia calon subjek penelitian , dan dilakukan pencatatan pada calon subjek penelitian yang hadir dengan mengisi daftar hadir. Secara berurutan para calon subjek penelitian diperiksa oleh beberapa dokter (tergantung jumlah calon subjek penelitian yang hadir) untuk pemeriksaan kelayakan seluruh calon subjek penelitian, Pemeriksaan ini untuk mengecek kriteria inklusi atau eksklusi termasuk anamnesis, pemeriksaan fisik, pemeriksaan status gizi dan pemeriksaan MMSE. Setelah dinyatakan masuk kriteria inklusi, calon subjek penelitian mendapat keterangan dari dokter tentang penelitian yang dilakukan, yang apabila setuju, lalu menandatangani inform concern yang telah diberikan.
3. Dilakukan *convenience random sampling* yaitu dengan cara sebagai berikut. Subjek penelitian yang telah menandatangani persetujuan penelitian itu

selanjutnya dikumpulkan, dicatat kembali dan diurutkan nomornya sesuai dengan kehadiran di depan petugas pencatat. Sebelum dibagi ke dalam 4 kelompok, dilakukan pengambilan darah subjek penelitian untuk pemeriksaan serologi antibodi influenza pertama (bulan ke 0 (nol)). Selanjutnya pihak ketiga melakukan randomisasi secara acak seluruh subjek penelitian ke dalam 4 kelompok penelitian dengan menggunakan *Microsoft Excel* dan diberikan intervensi sesuai kode yang telah diberikan oleh pihak ketiga tersebut, yaitu pemberian vaksin influenza atau plasebo vaksin influenza dan probiotik atau plasebo probiotik.

4. Di dalam penelitian ini menggunakan uji tersamar ganda, yakni di mana peneliti, pemeriksa laboratorium, dan partisipan tidak mengetahui siapa saja yang masuk di dalam kelompok intervensi dan plasebo.
5. Setelah subjek penelitian memenuhi kriteria sampel penelitian, peneliti melakukan prosedur intervensi pertama pada seluruh partisipan.
   A. Penyuluhan awal dan penandatanganan *Informed Consent*.
   B. Pengisian data penelitian awal.
   C. Pengambilan darah untuk pemeriksaan laboratorium pertama. Sampel darah ini diperiksa titer HI. Pihak pemeriksa laboratorium tidak mengetahui asal kelompok dari sampel darah yang diperiksa.
   D. Prosedur Intervensi:
      i. Pemberian vaksin atau plasebo vaksin influenza.
         1. Vaksin influenza atau plasebo disiapkan dalam spuit 1 cc, dari sediaan vial 0,5 cc tanpa pelarutan.
         2. Bagian tubuh yang dijadikan lokasi injeksi adalah deltoid sinistra, kecuali ditemukan kontra indikasi seperti luka terbuka. Lokasi injeksi dibersihkan dengan apus alkohol.
         3. Vaksin diinjeksikan secara intramuskular.
         4. Subjek penelitian diawasi selama 30 menit untuk reaksi simpang akut.
         5. Bila terjadi reaksi simpang akut, dilakukan tata laksana sesuai prosedur.

6. Seluruh subjek penelitian tidak dibolehkan mengkonsumsi probiotik selain yang diberikan oleh peneliti selama 6 bulan masa penelitian.

ii. Pemberian Probiotik atau Plasebo Probiotik.

1. Probiotik atau plasebo disiapkan dalam sediaan kapsul.
2. Subjek penelitian diminta untuk mengonsumsi probiotik atau plasebo pertama di hadapan peneliti.
3. Subjek penelitian diminta untuk mengonsumsi probiotik atau plasebo selanjutnya hingga enam bulan terakhir, yang diawasi oleh kader sambil melakukan pencatatan pada *logbook*.

E. Pencatatan pertama untuk prosedur intervensi.

6. Dalam waktu 6 bulan dilakukan tiga jenis pemantauan, yaitu:
   a. Pemantauan konsumsi probiotik atau plasebo probiotik.
   b. Pemantauan efek samping dari vaksinasi influenza (KIPI) dan probiotik.
   c. Pemantauan ILI (*influenza-like illness*)
   d. Partisipan yang mengalami efek samping intervensi dan ILI dilaporkan kepada peneliti untuk dilakukan diagnosis dan tatalaksana yang diperlukan. Peneliti meminta pihak ketiga, untuk melakukan diagnosis dan tatalaksana yang diperlukan, tetapi pihak tersebut tidak mengetahui asal kelompok dari partisipan yang diperiksa.
7. Pada subjek penelitian juga diberikan termometer untuk memeriksa suhu tubuh, *log book* untuk mencatat keluhan selama penelitian perlangsung, mencatat probiotik atau plasebo probiotik yang diminum selama 1 bulan ke depan. Pada reponden diberikan nomor kontak kader dan dokter yang dapat dihubungi selama 24 jam bila diperlukan, terutama bila ada keluhan demam dan batuk, pilek serta sakit tenggorokan.
8. Di pihak lain, peneliti menyiapkan 1 (satu) kader untuk sekitar 5 sd 15 subjek penelitian yang bertugas mengecek log book subjek penelitian, keadaan subjek penelitian dan ketaatan minum probiotik/plasebo probiotik yang telah diberikan setiap hari, dan melaporkan kepada dokter penanggung jawab bila terdapat keluhan pada pasien.

9. Bila ada keluhan dari subjek penelitian dokter penanggungjawab melakukan kunjungan rumah subjek tersebut, yang selanjutnya melakukan tidak lanjut bila diperlukan, seperti meresepkan obat dan/atau merujuk ke pelayanan kesehatan terdekat.
10. Para kader lalu mengisikan laporannya ke log book kader yang telah dibagikan, sesuai dengan jumlah subjek penelitian yang diawasinya.
11. Pemeriksaan logbook subjek penelitian, logbook kader dilakukan setiap bulan pada saat dilakukan pemeriksaan kesehatan subjek penelitian dan pemberian probiotik/plasebo.
12. Pada waktu 4 minggu setelah intervensi yang pertama dilakukan pemeriksaan darah untuk mengetahui titer HI yang ke dua. Selanjutnya dilakukan pemeriksaan setelah 4 bulan dari intervensi yang pertama.
13. Setelah 6 bulan intervensi, dilakukan prosedur pemeriksaan laboratorium ke-empat, untuk mengukur titer HI Influenza pascaintervensi.
14. Seluruh data terkait penelitian dikumpulkan untuk kemudian dilakukan analisis data.
15. Partisipan yang ikut serta di dalam penelitian ini didaftarkan asuransi BPJS kelas 3 dan ditanggung selama masa 6 bulan penelitian.

1.4 Alur Penelitian

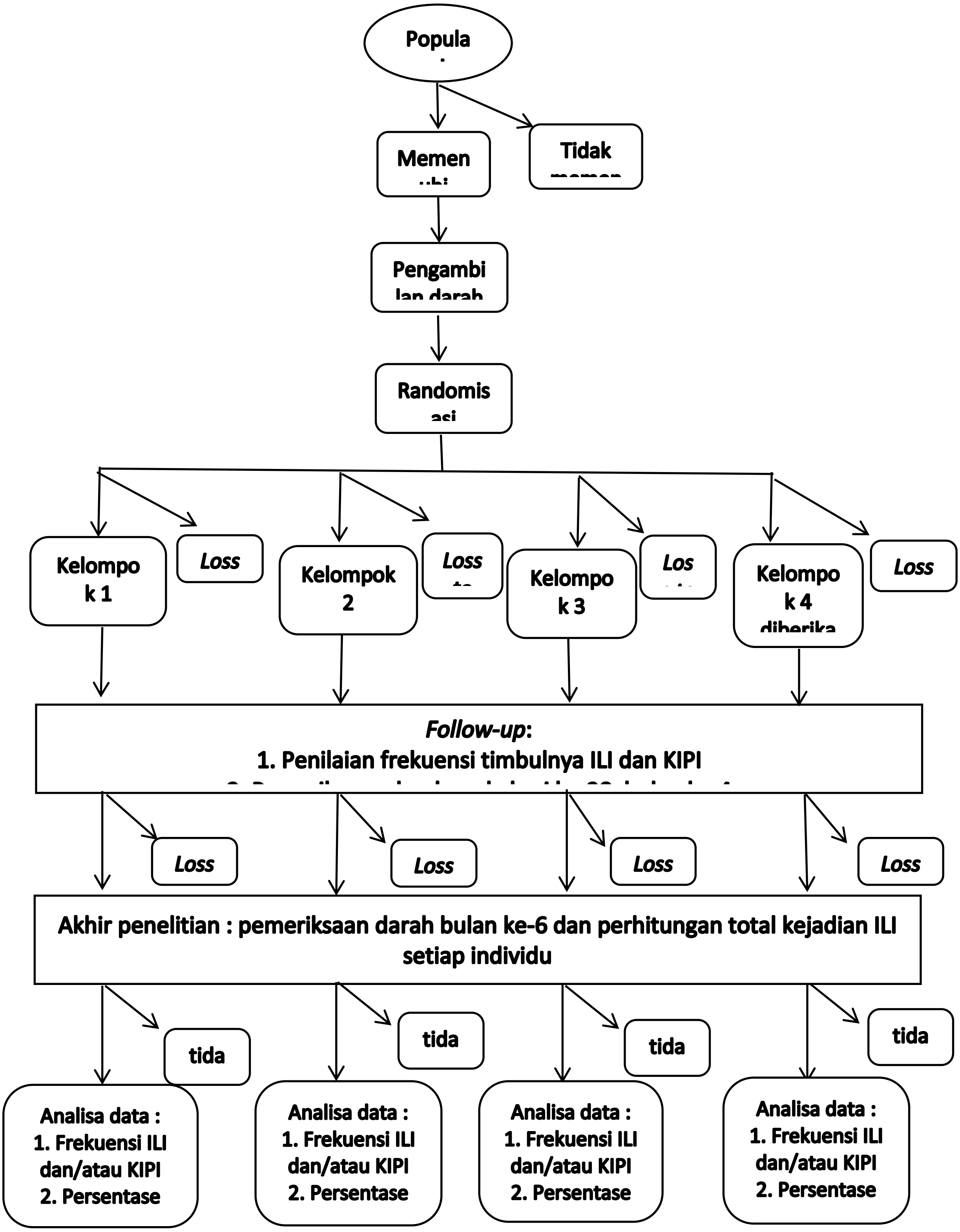

Gambar 4.1 Alur Penelitian

4.9 Manajemen dan Analisis Data

Setelah selesai pengumpulan data, selanjutnya dilakukan pengolahan data yang terdiri dari *editing, coding, tabulating, entri* data dan *cleaning*. Analisis statistik dilakukan setelah proses pengolahan data selesai. Analisis data yang digunakan dalam penelitian ini adalah:

a. Analisis univariat, untuk mendeskripsikan rerata, median, modus, proporsi, dll.
b. Uji homogenisitas, untuk melihat kesetaraan antara kelompok intervensi dan kelompok kontrol
c. Uji klinis, setelah intervensi dilakukan pengukuran variabel dependen pada semua subjek penelitian. Hasil pengukurannya dijadikan data untuk melakukan penghitungan *relative risk (RR), relative risk reduction (RRR),* yang menunjukkan berapa persen intervensi yang kita lakukan menurunkan angka kegagalan. Juga dihitung *absolute risk reduction (ARR),* yakni berapa perbedaan kegagalan faktual antara intervensi pemberian vaksin influenza dan pemberian probiotik dan *Number needed to treat (NNT),* yakni angka yang menyatakan berapa jumlah orang yang harus diberi intervensi dengan vaksin atau probiotik untuk mencegah 1 kasus atau menghindarkan 1 penderita ILI.

   Selain itu dinilai kemamputerapan hasil uji klinis, dengan menghitung keterjangkauan biaya dengan menghitung *cost of one treatment*, untuk mencegah satu outcome yang buruk (*one bad outcome*).

1.10 Keamanan Penelitian dan Penanganan Efek Samping

Seluruh kejadian efek samping dari penelitian ini dilaporkan langsung kepada peneliti untuk dilakukan pemeriksaan dan tatalaksana sesuai dengan kebutuhan.

Alur penanganan efek samping, kejadian pascaimunisasi dan ILI pada penelitian ini adalah sebagai berikut:

1. Subjek penelitian yang mengalami keluhan seperti demam, batuk, dan sesak, atau keluhan lain yang memerlukan tatalaksana medis diarahkan untuk memeriksakan diri ke fasilitas kesehatan yang telah ditunjuk oleh peneliti.
2. Tenaga kesehatan dan pendamping subjek penelitian diharuskan menghubungi peneliti untuk melaporkan kejadian tersebut. Tenaga kesehatan yang ditunjuk memutuskan apakah subjek penelitian bisa dilakukan rawat jalan, atau perlu dirujuk ke rumah sakit. Bila perlu dirujuk, subjek dirujuk di rumah sakit yang telah ditunjuk oleh peneliti.
3. Seluruh proses pengobatan, dari mulai perawatan hingga sembuh, pulang atau meninggal harus dilaporkan kepada peneliti oleh tenaga kesehatan pada fasilitas kesehatan yang telah ditunjuk.

4. Seluruh biaya yang diperlukan subjek penelitian untuk pengobatan efek samping/KIPI yang disebabkan oleh karena pemberian vaksin dan atau probiotik ditanggung oleh peneliti.
5. Pasien yang meninggal dalam periode penelitian akibat pemberian vaksin dan probiotik diberikan kompensasi yang sesuai.

4.11 Organisasi Penelitian

Peneliti utama : dr. Sukamto Koesnoe, SpPD

Pembimbing : dr. Asri C. Adisasmita, MPH, PhD.

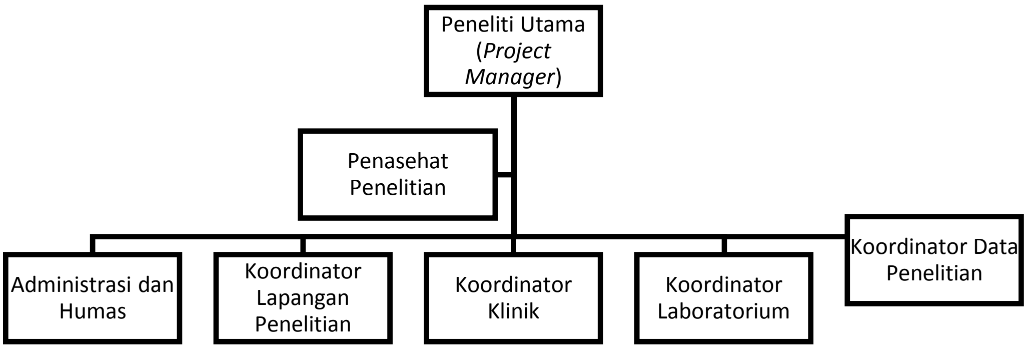

1.12 Masalah Etika

Pelaksanaan penelitian ini tunduk pada prinsip-prinsip ***“Deklarasi Helsinki”*** dan prinsip-prinsip yang digariskan dalam ***“Guideline for Good Clinical Practice”*** dari *ICH Tripartite Guideline* (ICH-GCP) maupun peraturan lokal yang berlaku di Indonesia. Penelitian ini mendapatkan surat lolos kaji etik *(ethical clearance)* dari Panitia Tetap Etik Penelitian. Kepada seluruh pasien sebagai subjek penelitian dan/atau keluarga diberikan penjelasan secara lisan dan tertulis mengenai tujuan dan prosedur penelitian, untuk kemudian dimintakan persetujuan tertulis untuk ikut serta dalam penelitian. Keikutsertaan pasien sebagai subjek penelitian ini didasarkan pada kesukarelaan. Pasien dan/ atau keluarga juga diberi kebebasan bila di tengah-tengah pelaksanaan penelitian kemudian memutuskan untuk menolak meneruskan penelitian.
