# Supplementary material for: A randomized controlled trial to evaluate the effect of influenza vaccination and probiotic supplementation on immune response and incidence of influenza-like illness in an elderly population in Indonesia
Source: PLoS One. 2021 Dec 16;16(12):e0250234. doi: 10.1371/journal.pone.0250234 (PMC8675694; doi:10.1371/journal.pone.0250234)
Supplement: S3 File — (PDF) [file pone.0250234.s003.pdf]

“Effect of Influenza Vaccination and Probiotic Supplementation on Immune Response and Incidence of Influenza-Like Illness in an Elderly Population”

Research Protocol

INTRODUCTION

Influenza is a major cause of mortality and morbidity worldwide. Indeed, influenza viruses can cause only minimal symptoms, but also can lead to severe and lethal complications. In general, influenza virus infections result in Acute Respiratory Illness (ARI). However, because ARI symptoms can also be caused by other infectious agents and are not specific to influenza viruses, this set of symptoms is referred to as Influenza-Like Illness (ILI). In Indonesia, there is currently no available report on the prevalence of this disease. Based on symptoms used to define ARIs, prevalence is estimated at 25%. Various studies have shown that influenza viruses and Respiratory Syncytial Virus (RSV) are often associated with acute respiratory disease requiring hospitalization, especially in the elderly population and patients with previous chronic disease. This is why individuals aged 65 years or older are considered among the most vulnerable groups, representing 90% of the reported cases of influenza-related complications.

Vaccination is considered as a primary preventive method in the management of influenza. The efficacy of a vaccine at preventing disease can be inferred based on its efficacy and effectiveness at inducing seroconversion, conferring seroprotection, and reducing ILI incidence. However, clinical studies on the effectiveness and efficacy of influenza vaccines in elderly populations have generated contradictory results.

Immunosenescence, which refers to the process of immune system aging that is reflected by an increased incidence of infections in the elderly, has been proposed as the cause underlying the reduced immunization response to vaccines observed in the elderly population. A new strategy is needed to improve the effectiveness of influenza vaccines in the elderly, either by improving the individuals’ immune response or vaccine formulations. In this study, we explore

whether probiotics can improve the immune response triggered by a trivalent influenza vaccine in the elderly, and reduce the incidence of ILI in this population.

Study Objectives: To investigate the effect of influenza vaccination with or without probiotic supplementation on the immune response and incidence of influenza-like illness (ILI) in the elderly.

Study Design

This study was a randomized, double-blind, placebo-controlled trial with a factorial design comparing the efficacy of two interventions, influenza vaccines and probiotics, at decreasing the risk of ILI in the elderly. Eligible participants were randomized into four intervention groups: 1) influenza vaccine + probiotics; 2) influenza vaccine + placebo; 3) placebo + probiotics; and 4) both placebo. This study was conducted in the entire Pulo Gadung District, East Jakarta, between April and December 2015, which was the period encompassing flu season.

1.1 Selection and Withdrawal Criteria

1.1.1 Inclusion Criteria

1. All elders who come to vaccination and health counseling activities held in all areas of the Health Center in East Jakarta District, are men and women aged ≥ 60 years.
2. In good health in the past 6 months, and in the initial procedure of the research.
3. BMI 17.5 - 29.9
4. Mental status with a mini mental state examination (MMSE) is considered healthy (MMSE score 28-30).
5. Good mobility.

1.1.2 Exclusion Criteria

1. Vaksin Influenza Contraindicated against influenza vaccine
   a. Severe allergy to chicken eggs or protein in chicken
   b. Have suffered from Guillain-Barre Syndrome

2. Currently undergoing treatment related to immune system modulation, for example:
   a. Intravenous immunoglobulin therapy in the past 4 weeks
   b. Immunosuppressant and/or corticosteroid therapy is equivalent to prednisone ≥ 20 mg/day, is being undertaken for more than 2 weeks, or only stopped less than 3 months before the research.
3. Have received influenza vaccination less than one year before.
4. Are taking probiotic agents, both in the form of a manufacturer or natural for more than 7 days.

1.1.3 Recruitment Procedure

1. Examination of participant eligibility

   With data on the number of reachable population in all areas of Health Center in East Jakarta. The researcher examines the eligibility of participants:

   i. Fill out the eligibility questionnaire.
   ii. Physical examination is required.
2. Acceptance of participants

   After the participants were selected, they were collected in each Integrated Service Post for Elderly (*Posyandu Lansia*) and given an explanation of the research.

3 . Participants who agree to participate in the research sign an informed consent.

1.1.4 Withdrawal or Drop Out Criteria

1. The patient dies.
2. The patient loses contact during observation with various causes, such as moving house.
3. Patients consciously and/or without influence say they want to quit the research.
4. Patients who receive immune modulation therapy or immunosuppressants during the research for any reason.

1.2 Laboratory Interventions and Examinations

1.2.1 Influenza Vaccine

Influenza vaccination using the *Flubio*® brand vaccine from Biofarma which is a Trivalent Inactivated Influenza Vaccine (TIV) contains haemagglutinin from influenza virus antigens, and has been made with a virus strain according to WHO recommendations for northern hemisphere in 2014-2015 (WHO, 2014) . The vaccine is in the form of clear suspension given for 0.5 ml IM injection in each selected research subject. Each dose contains influenza A/California/7/2009 (H1N1), A/Texas/50/2012 (H3N2), and B/Massachusetts/2/2012with hemagglutinin doses of 15 mg each.

1.2.2 Probiotics

The probiotics used in this study were 2 billion cfu organisms consisting of Lactobacillus acidophilus Rossel-52 and Lactobacillus rhamnosus Rosell-11, with additional substances: 211 mg maltodextrin, 8 mg magnesium stearate, 1 mg ascorbic acid. Physically the carrier is white, put in a clear manufacturer capsule with size number 2, with the name Lacidofil® which is distributed by PT Dexa Medica Probiotics is given 2 times per day, for 6 months continuously

1.2.3 Placebo

The vaccine control injected was placeboNaCl 0.9% 0.5 ml. Probiotic control is a placebo probiotic with the same size and color of the capsule as the probiotics, but does not cause a therapeutic effect. This probiotic placebo is a sterile trisi starch, which is packaged in clear capsule size 2.

1.2.4 Laboratory Examinations

Laboratory tests require the following materials and tools:

1. Hemagglutinin reagents for influenza virus serotypes H1N1, H3N2, and B according to the current circulating strain are twice the number of samples (for measurement of serum HI levels before and after vaccination).
2. Vaccutainer for storing blood and sending blood samples to the laboratory, twice the number of samples.
3. 3 cc syringes three times the number of samples to take blood samples before and after vaccination, and influenza vaccine injection.

4. Cotton alcohol for asepsis.
5. HI Influenza titer checking tool that has been calibrated by National Institute of Health Research and Development (*Litbangkes*).

1.3 Research Procedure

1. Data collection of the elders from the Integrated Service Post for Elderly (*Posyandu Lansia*) in the area of the Pulo Gadung District Health Center, East Jakarta. The data came from Integrated Service Post cadres under the guidance of the Kayu Putih Family Clinic, which are:
   a. Kayu Putih Integrated Service Post for Elderly
   b. Pisangan Timur 1 Integrated Service Post for Elderly
   c. Pisangan Timur 2 Integrated Service Post for Elderly
   d. Rawamangun Integrated Service Post for Elderly
   e. Cipinang Timur 1 Integrated Service Post for Elderly
   f. Cipinang Timur 2 Integrated Service Post for Elderly

2. The invitations were distributed to all Integrated Service Post for Elderly participants of prospective research subjects, and note taking on prospective research subjects present by filling in the attendance list. Sequentially, prospective research subjects were examined by several doctors (depending on the number of prospective research subjects present) for the feasibility examination of all prospective research subjects. This examination was to check the inclusion or exclusion criteria including history taking, physical examination, examination of nutritional status and MMSE examination. After being declared to be involved in the inclusion criteria, prospective research subjects received information from doctors about the research conducted, which if agreed, then signed the informed concern that had been given.

3. Convenience random sampling was carried out in the following way. Research subjects who have signed the research agreement were then gathered, recorded again and sorted according to their attendance in front of the registrar. Before being divided into 4 groups, blood samples were taken

for the study subjects for serological examination of the first influenza antibody (0th month (zero)). Then the third party randomized randomly all research subjects into 4 research groups using Microsoft Excel and was given an intervention according to the code given by the third party, namely the administration of influenza vaccine or placebo influenza vaccine and probiotic or probiotic placebo.

4. This research used a double-blind test, in which researchers, laboratory examiners, and participants did not know who were included in the intervention and placebo groups.

5. After the research subjects met the criteria of the study sample, the researcher conducted the first intervention procedure on all participants.
   A. Initial counseling and signing of the Informed Consent.
   B. Filling in the initial research data.
   C. Blood collection for the first laboratory examination. This blood sample was examined for HI titre. The laboratory examiner did not know the origin of the group from the blood sample examined.
   D. Prosedur Intervensi:
      i. Vaccination or placebo influenza vaccine.
   1. Influenza or placebo vaccines were prepared in a 1 cc syringe, from 0.5 cc vial preparation without dissolution.
   2. The body part used as the injection site was deltoid sinistra, unless contra indications are found such as open wounds. The injection site was cleaned with alcohol smear.
   3. Vaccines were injected intramuscularly.
   4. The study subjects were monitored for 30 minutes for acute adverse reactions.
   5. If an acute adverse reaction occurs, the procedure is carried out according to the procedure.
   6. All research subjects are not allowed to consume probiotics other than those given by researchers during the 6 month study period.

ii. Probiotics or Probiotic Placebo administration.

1. Probiotics or placebo were prepared in capsules.
2. Research subjects were asked to take probiotics or placebo first in in front of the researcher.
3. Research subjects were asked to consume probiotics or placebo further up to the last six months, which was monitored by cadres while taking notes on the logbook.

E. The first record for the intervention procedure.

6. Within 6 months three types of monitoring were carried out, which are:
    a. Monitoring the consumption of probiotics or placebo probiotics.
    b. Monitoring side effects from influenza vaccination (KIPI) and probiotics.
    c. ILI (influenza-like illness) monitoring
    d. Participants who experienced side effects of interventions and ILI were reported to the investigators for the necessary diagnosis and treatment. The researcher asks a third party, to make the necessary diagnosis and management, but the party does not know the origin of the group from the examinee.

7. The research subject was also given a thermometer to check body temperature, a log book to record complaints during ongoing research, record probiotics or placebo probiotics taken for the next 1 month. The respondent was given a cadre and doctor contact number that can be contacted for 24 hours if needed, especially if there are complaints of fever and cough, runny nose and sore throat.

8. On the other hand, the researcher prepares 1 (one) cadre for about 5 to 15 research subjects whose task is to check the research book's log book, the condition of the research subject and the probiotic/placebo observance of probiotics that have been given every day, and report to the physician in charge if there are complaints in patients.

9. If there is a complaint from the research subject, the responsible physician visits the subject's home, who then goes further if necessary, such as prescribing medication and/or referring to the nearest health service.

10. The cadres then fill out their reports into the cadres' log books that have been distributed, according to the number of research subjects they supervise.

11. Examination of research subjects' logbooks, cadre logbooks are conducted every month at the time of medical examination of research subjects and administration of probiotics placebo.

12. At 4 weeks after the first intervention a blood test is performed to determine the second HI titer. Furthermore, the examination was carried out after 4 months from the first intervention.

13. After 6 months of intervention, a fourth laboratory examination procedure is performed, to measure the post-intervention HI Influenza titer.

14. All research-related data were collected for data analysis.

15. Participants who participated in this study were registered Social Insurance Administration Organization (BPJS) class 3 insurance and were covered for a period of 6 months of research.

1.4 Research Flow

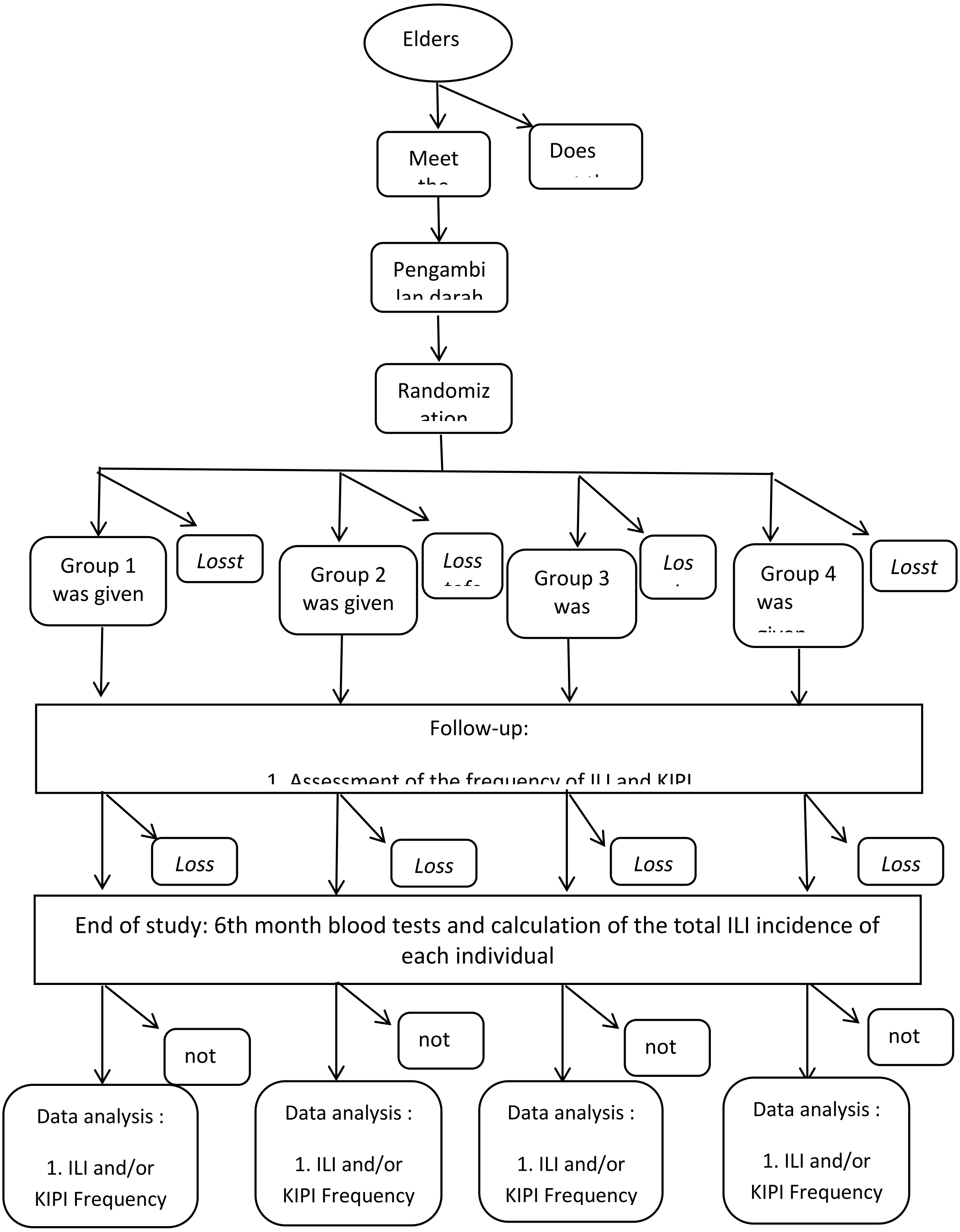

Figure 4.1 Research Flow

4.9 Data Management and Analysis

After completing data collection, data processing was performed which consists of editing, coding, tabulating, data entry and cleaning. Statistical analysis is done after the data processing is complete. Analysis of the data used in this study are:

a. Univariate analysis, to describe the mean, median, mode, proportion, etc.
b. Homogenisity test, to see the equality between the intervention group and the control group
c. Clinical trials, after the intervention, the measurement of the dependent variable was carried out on all research subjects. The measurement results are used as data to calculate the relative risk (RR), relative risk reduction (RRR), which shows what percentage of interventions we do reduce the failure rate. Also calculated absolute risk reduction (ARR), ie what is the difference in factual failure between influenza vaccine intervention and probiotic administration and Number needed to treat (NNT), which is a number that states how many people should be intervened with vaccines or probiotics to prevent 1 case or avoid 1 ILI sufferer.

   Besides, the ability of clinical trial results was assessed, by calculating the affordability of costs by calculating the cost of one treatment, to prevent one bad outcome.

1.10 Safety of Research and Handling of Side Effects

All adverse events from this research were reported directly to the researcher for examination and treatment as needed.

The flow of handling of side effects, post-immunization events, and ILI in this research are as follows:

1. Research subjects who experience complaints such as fever, cough, and spasms, or other complaints that require medical treatment are directed to check themselves into health facilities that have been designated by researchers.
2. Health workers and research subject companions are required to contact the researcher to report the incident. The designated health worker decides whether the research subject can be done outpatient, or needs to be referred to a hospital. If it is necessary to be referred, the subject is referred to the hospital appointed by the researcher.
3. The entire treatment process, from treatment to recovery, discharge or death, must be reported to the researcher by health personnel at the designated health facility.

4. All costs required by research subjects for the treatment of side effects / KIPI caused by the administration of vaccines and/or probiotics are borne by the researcher.
5. Patients who died in the research period due to vaccines and probiotics were given appropriate compensation.

4.11 Research Organization

The Researcher : dr. Sukamto Koesnoe, SpPD

Supervisor : dr. Asri C. Adisasmita, MPH, PhD.

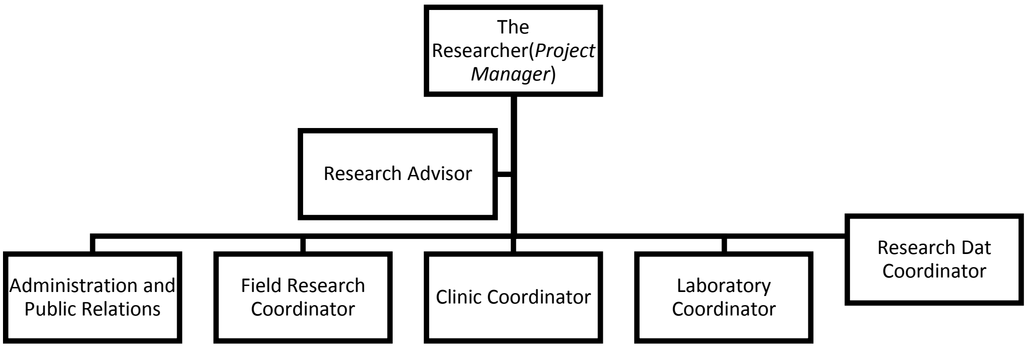

1.12 Ethical Issues

The implementation of this research is subject to the principles of the "***Declaration of Helsinki***" and the principles outlined in the "***Guidelines for Good Clinical Practice***" of the ICH Tripartite Guidelines (ICH-GCP) as well as local regulations that apply in Indonesia. This study received a letter of ethical clearance (ethical clearance) from the Permanent Research Ethics Committee. All patients as subjects of the study and or family are given an oral and written explanation of the aims and procedures of the study, and then written consent is requested to participate in the study. The participation of patients as the subjects of this study is based on volunteerism. Patients and/or families are also given freedom if in the midst of conducting research then decides to refuse to continue research
